# Cyclic‐di‐GMP induces inflammation and acute lung injury through direct binding to MD2

**DOI:** 10.1002/ctm2.1744

**Published:** 2024-08-21

**Authors:** Chenchen Qian, Weiwei Zhu, Jiong Wang, Zhe Wang, Weiyang Tang, Xin Liu, Bo Jin, Yong Xu, Yuyang Zhang, Guang Liang, Yi Wang

**Affiliations:** ^1^ School of Pharmacy Hangzhou Normal University Hangzhou Zhejiang China; ^2^ Chemical Biology Research Center School of Pharmaceutical Sciences Wenzhou Medical University Wenzhou Zhejiang China; ^3^ School of Pharmaceutical Sciences Hangzhou Medical College Hangzhou Zhejiang China

**Keywords:** acute lung injury, COVID‐19, cyclic‐di‐GMP, MD2

## Abstract

**Background:**

Severe bacterial infections can trigger acute lung injury (ALI) and acute respiratory distress syndrome, with bacterial pathogen‐associated molecular patterns (PAMPs) exacerbating the inflammatory response, particularly in COVID‐19 patients. Cyclic‐di‐GMP (CDG), one of the PAMPs, is synthesized by various Gram‐positve and Gram‐negative bacteria. Previous studies mainly focused on the inflammatory responses triggered by intracellular bacteria‐released CDG. However, how extracellular CDG, which is released by bacterial autolysis or rupture, activates the inflammatory response remains unclear.

**Methods:**

The interaction between extracellular CDG and myeloid differentiation protein 2 (MD2) was investigated using in vivo and in vitro models. MD2 blockade was achieved using specific inhibitor and genetic knockout mice. Site‐directed mutagenesis, co‐immunoprecipitation, SPR and Bis‐ANS displacement assays were used to identify the potential binding sites of MD2 on CDG.

**Results:**

Our data show that extracellular CDG directly interacts with MD2, leading to activation of the TLR4 signalling pathway and lung injury. Specific inhibitors or genetic knockout of MD2 in mice significantly alleviated CDG‐induced lung injury. Moreover, isoleucine residues at positions 80 and 94, along with phenylalanine at position 121, are essential for the binding of MD2 to CDG.

**Conclusion:**

These results reveal that extracellular CDG induces lung injury through direct interaction with MD2 and activation of the TLR4 signalling pathway, providing valuable insights into bacteria‐induced ALI mechanisms and new therapeutic approaches for the treatment of bacterial co‐infection in COVID‐19 patients.

## Graphical abstract

1. CDG mediates lung injury through direct interaction with MD2 at isoleucine residues at positions 80 and 94, along with phenylalanine at position 121.

2. MD2 blockade using a specific inhibitor or genetic knockout attenuates CDG‐induced ALI via reducing TLR4‐mediated inflammation.

3. CDG induces inflammation by inducing TLR4/MD2 complex formation and further activates both MyD88‐ and TRIF‐dependent cascades.

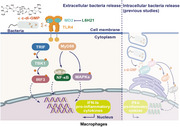

## 1 INTRODUCTION

Acute lung injury (ALI) and its more severe form, acute respiratory distress syndrome (ARDS) with high mortality, are characterized by lung neutrophil infiltration, diffuse alveolar damage, ventilation and blood flow imbalance, acute pulmonary inflammation, and lung damage.^1^, ^2^ Bacterial infections lead to pneumonia or sepsis, causing severe inflammatory damage to the lungs, and subsequently leading to the development of ALI or ARDS in patients.^3^, ^4^ Recent reports have demonstrated that bacterial co‐infection in patients with Coronavirus disease 2019 (COVID‐19) is an important driver of mortality.^5^, ^6^, ^7^ Elevated levels of bacterial‐derived pathogen‐associated molecular patterns (PAMPs), such as lipopolysaccharide (LPS) and bacterial DNA, are present in the serum and endotracheal aspirates of COVID‐19 ICU patients.^8^ Thus, understanding the pathogenic factors of acute respiratory bacterial co‐infection is vital for treating COVID‐19 patients and ensuring the responsible use of antibiotics.

However, aside from the widely studied LPS, a prominent component of the outer cell wall of Gram‐negative bacteria, there are few studies concerning other bacterial‐derived PAMPs such as flagellin, peptidoglycan, cyclic‐di‐GMP (CDG), and 3,4 lipoteichoic acid.^4^, ^9^, ^10^, ^11^, ^12^ Among these PAMPs, CDG is primarily synthesized by various Gram‐positive and Gram‐negative bacteria, including members of the Enterobacteriaceae family like *Escherichia coli* and *Salmonella*, as well as other species such as *Pseudomonas* and *Shewanella*. Studies suggest that levels of CDG are elevated in patients with certain pulmonary diseases, such as pneumonia, ARDS, and ventilator‐associated pneumonia.^13^, ^14^, ^15^ In addition, CDG is a potent immunomodulator, sensed by host immune proteins such as the stimulator of interferon genes (STING), leading to the production of potent inflammatory and antiviral cytokines, notably type I interferon.^16^, ^17^, ^18^ Since severe bacterial infections can trigger ALI through bacterial PAMPs exacerbating the inflammatory response, CDG might play a potential role in pulmonary inflammation and infection.

CDG is not cell‐permeable due to the presence of two negatively charged phosphate groups in its structure, indicating CDG accumulation inside host cells occurs via active transport.^19^, ^20^ So far, previous studies have mainly focused on the process of intracellular bacteria‐released CDG into the host's cytosol by efflux pumps or bacteria autolyzes, leading to the cytosolic DNA‐mediated inflammatory responses in host cells.^18^, ^19^, ^21^ Interestingly, McWhirter and his group first found that overlapping CDG at higher concentrations can induce IFN‐β expression.^19^ Wang et al.^22^ further proved that CDG increases cyclooxygenase‐2 (COX‐2) secretion in macrophages in a STING‐independent manner. These studies suggest that extracellular CDG might induce inflammatory response via pattern recognition receptors (PRRs). However, the molecular mechanisms by which the released CDG associates with innate immune responses when extracellular bacteria autolyze or rupture remain unknown.

In this study, we evaluated the role of extracellular CDG in ALI and further investigated how extracellular CDG activates the inflammatory response in the lungs through PRRs, bypassing cytoplasmic DNA sensors. Our data showed that extracellular CDG directly interacted with myeloid differentiation protein 2 (MD2), a novel CDG receptor, leading to activation of TLR4‐mediated inflammatory response and subsequent lung injury. Our research provides valuable insights into the mechanisms by which bacteria can induce ALI and suggests new therapeutic approaches for the treatment of bacterial co‐infection in COVID‐19 patients.

## 2 MATERIALS AND METHODS

### 2.1 Reagents

CDG with >98% purity was purchased from MedChemExpress (HY‐107780B). CDG was dissolved in sterile water for in vitro studies or in 0.9% saline solution for in vivo studies. L6H21 was synthesized and structurally identified using MS and 1H NMR analyses as described in our previous paper.^23^ Bovine serum albumin (BSA) was purchased from Sigma‐Aldrich. Recombinant human MD2 (rhMD2; 1787‐MD‐050/CF) protein was obtained from R&D Systems. Antibodies against p38 (9212S), phosphorylated (p‐) p38 (Thr180/Tyr182, 9211S), c‐Jun N‐terminal kinase (JNK, 9252S), phosphorylated (p‐) JNK (Thr183/Tyr185, 4668S), extracellular signal‐regulated kinase (ERK, 4695S), phosphorylated (p‐) ERK (Thr202/Tyr204, 4370S), glyceraldehyde‐3‐phosphate dehydrogenase 1 (GAPDH, 5174S), TANK binding protein 1 (TBK1, 38066S), phosphorylated (p‐) TBK1(Ser172, 5483S), phosphorylated (p‐) IRF3 (Ser396, 4947S), IRF3 (4302S), myeloid differentiation primary response 88 (MyD88, 4283S), intercellular adhesion molecule‐1 (ICAM‐1, 26104SF), STING (13647S) and inhibitor of κB‐α (IκB‐α, 4812S) were from Cell Signaling Technology. Antibodies against TLR4 (sc‐293072), MD2 (sc‐80183), and macrophage markers CD68 (sc‐20060) were purchased from Santa Cruz Biotechnology. Toll/IL‐1R domain‐containing adaptor‐inducing IFN‐beta (TRIF, ab13810) was purchased from Abcam. Interleukin‐6 (IL‐6) antibody (66146‐1‐lg) was purchased from Proteintech. Vascular cell adhesion molecule‐1 (VCAM‐1, 39036S) was from Cell Signaling Technology. HRP‐linked second Antibodies (A0216, A0208) were from purchased Beyotime. Assay kits for MPO were purchased from Nanjing Jiancheng Bioengineering Institute. Beef extract (G8270‐500 g) was purchased from Solarbio. Tryptone (LP0042) was purchased from OXOID.

### 2.2 Animals

Male C57BL/6J (WT) mice, aged 8 weeks and weighing between 18−22 g, were procured from the Animal Center of Wenzhou Medical University. Age‐matched male global *Md2* ^−/‐^ (B6.129P2‐Ly96 KO; MD2KO) mice on a C57BL/6J background were sourced from the Riken BioResource Center. Male global *Tlr4* ^−/−^ (TLR4KO) mice on a C57BL/6J background (strain number: 029015) were obtained from Jackson Laboratory. All animals were housed in a pathogen‐free environment at 22°C ± 2°C, with 50%−60% humidity, under a 12 h light/dark cycle, and provided with a standard rodent diet and water. Ethical approval for all animal care and experimental procedures was obtained from the Animal Policy and Welfare Committee of Wenzhou Medical University (approval number: wydw2022‐0312). Animals were allowed a 2‐week acclimatization period in the laboratory prior to commencing the studies.

### 2.3 Cell isolation and culture

The HEK‐293T (human embryonic kidney epithelial) cell line was obtained from the Shanghai Institute of Cell Biology of the Chinese Academy of Sciences. HEK‐293T cells were cultured in Dulbecco's modified Eagle's medium (DMEM, C11995500BT, Gibco) supplemented with 10% fetal bovine serum (FBS, 10099−141, Gibco) and 1% penicillin/streptomycin (BC‐CE‐007, Biochannel).

Mouse primary peritoneal macrophages (MPMs) were isolated from MD2KO, TLR4KO mice, or WT mice. MPMs isolation involved intraperitoneal injection with 6% thioglycolate solution (0.03 g beef extract, 0.1 g tryptone, and 0.05 g NaCl dissolved in 10 mL double‐distilled water and filtered through a 0.22 µm filter; 3 mL per mouse), followed by 48 h of pathogen‐free housing. Afterwards, total MPMs were collected by peritoneal cavity washing with 5 mL RPMI‐1640 medium (C11875500BT, Gibco) per mouse. The harvested cells were centrifuged and resuspended in RPMI‐1640 medium supplemented with 10% FBS and 1% penicillin/streptomycin. MPMs were seeded at a density of 7 × 10^5^ cells per 35 mm diameter well and used 24 h after plating. Non‐adherent cells were eliminated by washing with a medium at 2 h postseeding.

### 2.4 Animal modeling of ALI and MD2 inhibitor treatment

To develop a CDG‐induced ALI model, WT and MD2KO mice were subjected to intratracheal injection of CDG (3 mg/kg, dissolved in 0.9% saline solution) or 0.9% saline solution alone. The experimental groups comprised (*n* = 6 per group): (1) WT mice treated with vehicle control (sham + WT), (2) CDG‐challenged WT mice (CDG + WT), (3) MD2KO mice treated with vehicle control (sham + KO), and (4) CDG‐challenged MD2KO mice (CDG + KO). At 6 h post‐CDG challenge, mice were euthanized under anaesthesia, and bronchoalveolar lavage fluid (BALF) was collected by perfusing 4 × 200 µL phosphate‐buffered saline (PBS) solution through the tracheal cannula. Blood and lung tissue samples were then collected for further analysis.

For the MD2 inhibitor‐treated ALI model, L6H21, a specific MD2 inhibitor, was used to confirm the role of MD2 in CDG‐induced ALI. Male C57BL/6J mice were randomly allocated into 5 groups (*n* = 6 per group): (1) mice treated with vehicle control (Ctrl), (2) mice treated with 10 mg/kg L6H21 (L6H21 10), (3) CDG‐challenged mice (CDG), (4) CDG‐challenged mice treated with 5 mg/kg L6H21 (CDG + L6H21 5), and (5) CDG‐challenged mice treated with 10 mg/kg L6H21 (CDG + L6H21 10). L6H21 was dissolved in a 3% dimethyl sulfoxide (DMSO) solution (diluted in corn oil) and intraperitoneally administered at a volume of 10 µL/g body weight every 12 h for 36 h prior to the CDG challenge. The Ctrl and CDG groups received the same volume of 3% DMSO solution (diluted in corn oil). At 6 h post‐CDG challenge, mice were euthanized under anesthesia, and BALF, blood, and lung tissue samples were collected as described above.

Pulmonary oedema was assessed by the ratio of wet weight to dry weight of the upper lobe right lung (wet/dry ratio). First, the wet weight of the upper lobe right lung is measured (wet weight), followed by drying the lung tissue at 62°C for over 48 h and then reweighing (dry weight). BALF samples were collected from the left lungs, and serum samples were utilized for measuring IL‐6 and tumour necrosis factor‐alpha (TNF‐α) levels by enzyme‐linked immunosorbent assay (ELISA). Additionally, BALF samples were used for cell counting.

To assess survival rates, the experimental groups consisted of (*n* = 8 per group): (1) WT mice treated with vehicle control (sham); (2) CDG‐challenged WT mice (CDG); (3) CDG‐challenged MD2KO mice (CDG + MD2KO); and (4) CDG‐challenged mice treated with 10 mg/kg L6H21 (CDG + L6H21). Mice mortality was monitored every 12 h starting from the CDG injection for 72 h. After 72 h post‐CDG injection, surviving mice were euthanized under anesthesia. The euthanasia procedure adhered to institutional ethical committee regulations.

### 2.5 Lung histology and immunostaining

After fixation in 4% paraformaldehyde and embedding in paraffin, lung tissue samples were sectioned at a thickness of 5 µm. The sections were stained with hematoxylin and eosin (H&E) following the manufacturer's instructions (G1120, Solarbio). Images were acquired using a bright field microscope (×400, Nikon TE2000). Lung injury scores (0‐1) were assessed based on published guidelines (Supporting information Table S1).^24^ The final injury scores were calculated using the formula: Score = [(20 × a) + (14 × b) + (7 × c) + (7 × d) + (2 × e)]/(number of fields × 100).

Immunoreactivity was detected using 3,3′‐diaminobenzidine (DAB). Lung sections were deparaffinized, rehydrated, and blocked in 5% BSA in PBS for 30 min at 37°C. Primary antibodies (1:100) were applied overnight at 4°C, followed by incubation with horseradish peroxidase‐labeled secondary antibodies for 2 h at 37°C. Nuclear staining was performed with DAB for 2 min at room temperature. Images were captured using a bright field microscope (×400, Nikon TE2000).

### 2.6 Myeloperoxidase activity assay

Lung tissue samples were assessed for MPO activity using an MPO Detection Kit (A044‐1‐1, Nanjing Jiancheng Bioengineering Institute) to evaluate neutrophil tissue infiltration, following the manufacturer's instructions.

### 2.7 Wright‐Giemsa staining

Each BALF liquid sample was centrifuged at 300 × *g* for 10 min in a cytocentrifuge to attach the cells to a slide. Subsequently, the slide was stained using a Wright‐Giemsa Stain Kit (ab245888, Abcam), following the manufacturer's instructions.

### 2.8 ELISA assay for cytokines

The protein levels of IL‐6 and TNF‐α in mouse serum and BALF samples were quantified using ELISA kits (Thermo Fisher Scientific), following the manufacturer's recommendations. The data were normalized to total proteins in the respective cell lysates.

### 2.9 Western blot and co‐immunoprecipitation

Total protein was extracted from cell or lung tissue lysates and then separated on 10% sodium dodecyl sulfate (SDS)‐acrylamide gels before being transferred onto polyvinylidene fluoride membranes. The membranes were blocked with skim milk for 1 h at room temperature, followed by overnight incubation with primary antibodies at 4°C. Subsequently, secondary antibodies were applied for 2 h at room temperature.

MD2‐TLR4 interactions were assessed via co‐immunoprecipitation. Protein lysates were incubated with TLR4 antibody at 4°C overnight. Subsequently, the protein lysates were precipitated using protein A + G agarose beads (P2012, Beyotime), and utilized for the detection of interacting proteins through immunoblotting. HRP‐linked second Antibodies (ab157532, ab131368) were purchased from Abcam. Immunoreactivity was visualized using a ChemiDoc XRS + system (Bio‐Rad) following the addition of an enhanced chemiluminescence reagent. Band densities were quantified using Image J software.

For immunoprecipitation, cell extracts were prepared and incubated with anti‐MD2 antibody (1:200) for 1 h. Immunoprecipitation was then carried out using protein G‐sepharose beads at 4°C overnight.

### 2.10 Real‐time reverse transcriptase‐polymerase chain reaction assay

Total RNA was extracted from cells and lung tissues using Trizol (15596018, Invitrogen). The isolated RNA was reverse‐transcribed using PrimeScript RT reagent Kit (9762, TaKaRa). Real‐time reverse transcriptase‐polymerase chain reaction (RT‐qPCR) was conducted using the Eppendorf Real plex 4 instruments (Eppendorf,). Primers for genes includin*g interleukin‐1 beta (Il1b*), *Il6*, *Tnf*, interferon‐stimulated gene 15 (*Isg15*), interferon‐beta gene (*Ifnb1*), and *Actb* were synthesized and obtained from Sangon Biotech. The primer sequences for all genes are provided in Supporting information Table S2. The relative mRNA levels of each gene were normalized to the levels of *Actb*.

### 2.11 Surface plasmon resonance assay

The binding affinity of CDG to rhMD2, TLR4, and TLR2 proteins was assessed using the Biacore T200 instrument (Cytiva) with a CM5 sensor chip (Cytiva). Briefly, rhMD2, TLR4, or TLR2 proteins were immobilized onto the sensors using an Amine coupling kit (Cytiva). CDG samples with varying concentrations were prepared in a running buffer (PBS with 0.5% P20 and 5% DMSO). Six concentrations were sequentially injected at a flow rate of 30 µL/min for a 200‐s association phase, followed by a 100‐s dissociation phase at 25°C. The obtained data were analyzed using Biacore T200 software EV. The Kd values were determined through global fitting of the kinetic data obtained from different CDG concentrations using the 1:1 Langmuir binding model.

### 2.12 Bis‐ANS displacement assay

Bis‐ANS (4,4′‐Bis(phenylamino)—[1,1′‐binaphthalene]−5,5′‐disulfonic acid dipotassium salt, 5 µM, Tocris Bioscience) and rhMD2 (5 nM) were mixed in PBS (pH 7.4), and different concentrations of CDG were added. After a 15 min incubation, the relative fluorescence units emitted at 430−590 nm were measured using a SpectraMax M5 Multi‐Mode Microplate Reader at 25°C.

### 2.13 Molecular docking of CDG to MD2

The crystal structure of human MD2 (PDB code: 2E56) was acquired from the Protein Data Bank repository. Input files for ligand‐receptor docking were prepared using the Graphical User Interface program AutoDock Tools 1.5.6 (Scripps Research). Molecular docking was conducted using AutoDock Vina 1.0.2 (Scripps Research). The top 30 conformations of the CDG‐MD2 docking results were selected for further analyses. Binding free energy was calculated using the MM/GBSA method in the AmberTools package following structural minimization. Finally, key residues involved in protein‐ligand interactions were identified based on per‐residue decomposition energy calculations.

### 2.14 MD2 mutation and expression

The MD2^WT^ and MD2 mutation (MD2^Mut^, I80A/I94A/F121A) plasmids were synthesized by Genechem. HEK‐293T cells and MPMs derived from MD2KO mice were transfected with either plasmids or empty vectors. Transfection was performed using LipofectAMINE™ 3000 (L3000015, Thermo Fisher Scientific).

### 2.15 siRNA‐induced gene silencing

Gene expression was silenced using the siRNA technique, with STING siRNA obtained from RiboBio. Transfection of MPMs with siRNA was conducted using LipofectAMINE 3000 (L3000015, Thermo Fisher Scientific), following the manufacturer's instructions. The target sequences were as follows: siSTING: 5′‐GGATCCGAATGTTCAATCA‐3′.

### 2.16 Statistical analysis

Data are presented as mean ± SEM. Image quantification was conducted using ImageJ software. Statistical analyses were performed using GraphPad Prism 7.0 software (version 7.0). Differences between the two groups were assessed using a two‐tailed Student's *t*‐test. For comparisons among multiple groups, one‐way ANOVA followed by Dunnett's multiple comparisons test was utilized. Survival data was analyzed by log‐rank (Mantel‐Cox) test. Detailed statistical information including *p*‐values and sample sizes (*n*) can be found in figure legends and expanded figure legends. Animal studies were conducted in a blinded manner, and no animals were excluded from the study. The sample size for each study was determined based on our experience with previous studies employing ALI animals and knockout mice in our laboratory.

## 3 RESULTS

### 3.1 CDG activates the MD2‐TLR4 signalling pathway in macrophages

**FIGURE 1:**
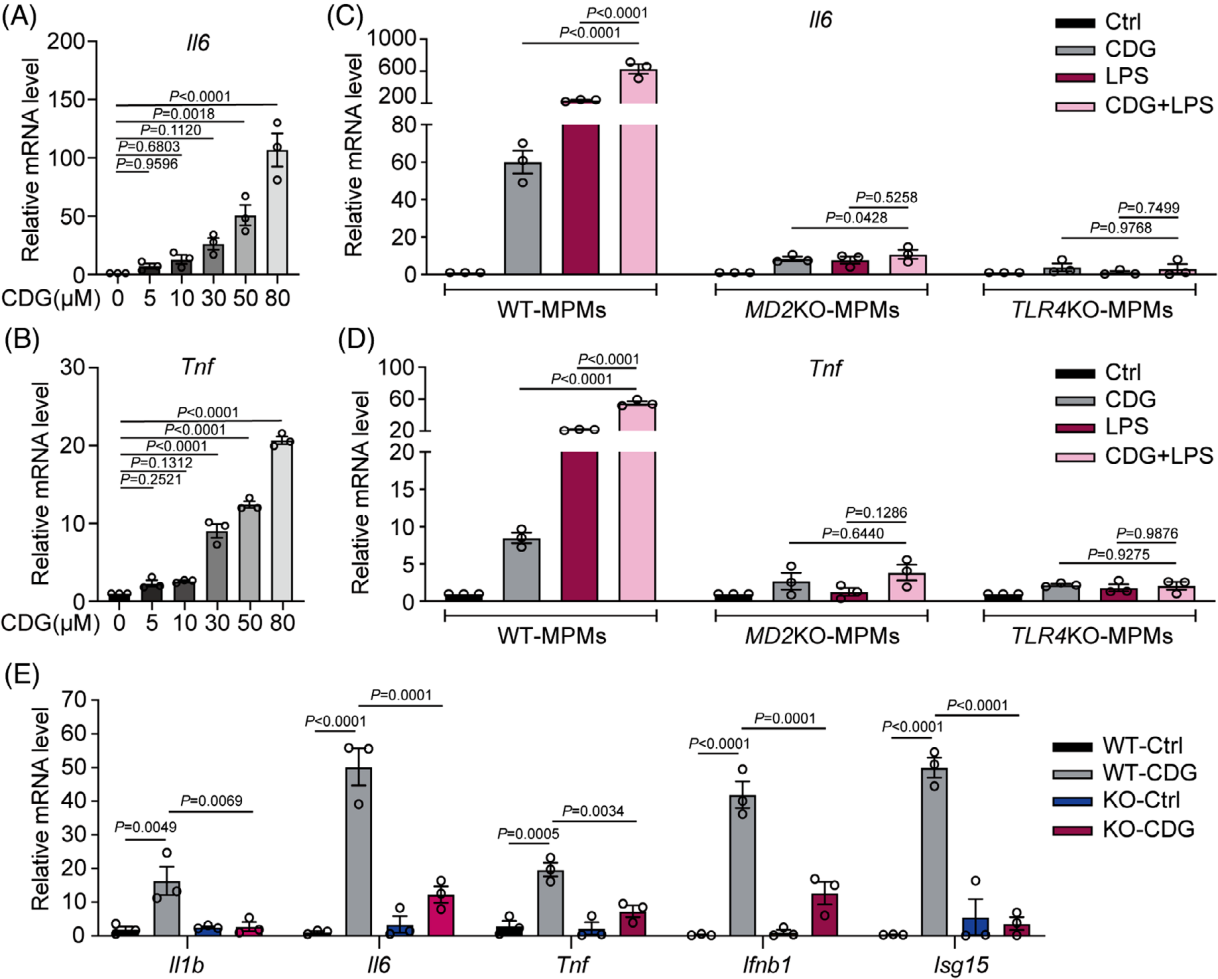
CDG‐induced inflammatory response in macrophages via MD2. (A, B) Mouse primary macrophages (MPMs) derived from C57BL/6J (WT) mice were treated with different concentrations of CDG for 12 h. The mRNA levels of *Il6* (A) and *Tnf* (B) were measured via RT‐qPCR assay. Data were normalized to the levels of *Actb* (*n* = 3 in each group, biological replicates). (C, D) MPMs were derived from WT, global *Md2* ^−/−^ (MD2KO), or global *Tlr4* ^−/−^ (TLR4KO) mice, respectively. MPMs were treated with CDG (80 µM), LPS (0.5 µg/mL), or CDG (80 µM) combined with LPS (0.5 µg/mL), and then the mRNA levels of *Il6* (C) and *Tnf* (D) were measured via RT‐qPCR assay. Data were normalized to the levels of *Actb* (*n* = 3 in each group, biological replicates). E MPMs derived from WT and MD2KO mice were treated with CDG (80 µM) for 12 h. The mRNA levels of *Il1b*, *Il6*, *Tnf*, *Ifnb1*, and *Isg15* were measured via RT‐qPCR assay. Data were normalized to the levels of *Actb* (*n* = 3 in each group, biological replicates). Data information: Data are presented as mean ± SEM. One‐way ANOVA followed by Dunnett's multiple comparisons test.

Emerging evidence revealed that macrophages, including resident alveolar macrophages and recruited macrophages from the bloodstream, play critical roles in the pathogenesis of ALI/ARDS. These macrophages release cytokines that recruit neutrophils or monocytes, thereby promoting and sustaining inflammation.^25^ Thus, we evaluated the ability of CDG to activate immune/inflammatory responses in MPMs. Similar to its effect on inducing inflammatory responses in host cells,^19^ we found CDG increased transcription of *Il6* and *Tnf* dose‐dependently in MPMs, indicating that CDG was able to stimulate inflammation as an extracellular inducer (Figure 1A and B). Studies suggest that TLR4 signalling plays an essential role in mediating lung injury in ALI and ARDS, and therapeutic drugs and methods targeting TLR4/TLR4‐accessory MD2 have effective therapeutic effects in the treatment of ALI and ARDS.^4^, ^26^, ^27^ Based on the important role of the TLR4 signalling pathway in sensing the bacterial‐derived PAMPs, we assumed that TLR4 signalling might play an important role in CDG‐induced inflammation. Thus, we explored the potential role of TLR4 or TLR4‐accessory protein, MD2, in CDG‐induced inflammation. The expression levels of *Il6* and *Tnf* were assessed following stimulation with CDG alone‐, LPS alone (the classic TLR4 agonist)‐ or CDG combined with LPS‐stimulation (CDG + LPS) in WT‐, MD2KO‐ or TLR4KO‐MPMs. Our results showed that CDG elevated inflammatory cytokines, less than LPS, and showed synergetic effects with LPS in WT‐MPMs (Figure 1C and D). Either MD2KO or TLR4KO significantly reduced CDG or CDG + LPS‐caused release of inflammatory cytokines (Figure 1C and D). All these findings suggest the involvement of the TLR4 signalling pathway, particularly MD2, in CDG‐induced inflammation. Next, we further explored the impact of MD2 deficiency on CDG‐induced inflammation in MPMs. As shown in Figure 1E, the expression levels of inflammatory cytokines, such as *Il1b*, *Il6*, *Tnf*, *Ifnb1*, and *Isg15*, were elevated upon CDG stimulation, while MD2 deficiency significantly reversed these changes in CDG‐treated MPMs. All these data suggest that the CDG‐induced inflammatory response in MPMs was significantly related to MD2.

**FIGURE 2:**
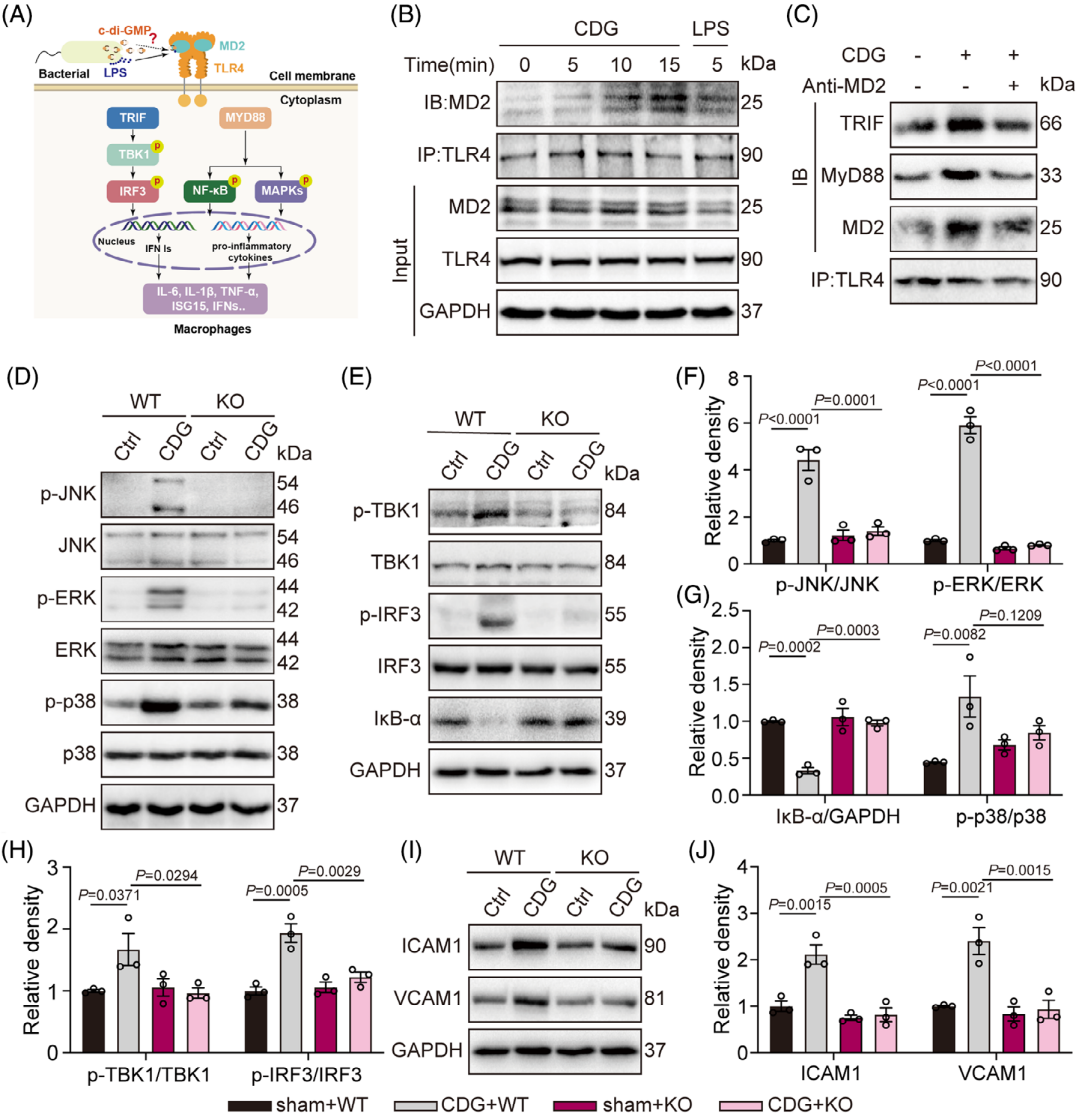
CDG mediated both the MyD88‐ and TRIF‐dependent pathways in MPMs. (A) Schematic diagram showing the MD2‐TLR4 signalling pathway and downstream MyD88‐ and TRIF‐dependent branches. (B) MPMs from WT mice were stimulated with CDG (80 µM) for 5, 10, or 15 min. Representative immunoblots of co‐immunoprecipitation of MD2 and TLR4 in MPMs were shown. LPS (0.5 µg/mL, 5 min) was used as the positive control. (C) MPMs derived from WT mice were stimulated with CDG (80 µM) for 15 min. MD2 blockade was achieved using MD2‐neutralizing antibody (anti‐MD2, 100 ng/mL). Representative co‐immunoprecipitation images of TRIF/TLR4/MD2 and MyD88/TLR4/MD2 were shown. (D–H) MPMs from WT or MD2KO mice were treated with CDG (80 µM) for 1 h. Activation of MyD88‐ and TRIF‐dependent pathways was measured. Unphosphorylated proteins and/or GAPDH were used as the loading controls (*n* = 3 in each group, biological replicates). Representative blots (D, E) and densitometric quantification (F–H) are shown. (I, J) MPMs from WT mice were stimulated with CDG (80 µM) for 24 h. The protein levels of ICAM1 and VCAM1 were examined via Western blot analysis. GAPDH was used as the loading control (*n* = 3 in each group, biological replicates). Representative blots (I) and densitometric quantification are shown (J). Data information: Data are presented as mean ± SEM. One‐way ANOVA followed by Dunnett's multiple comparisons test.

The classic TLR4 signalling pathway, including MyD88‐ and TRIF‐dependent branches, is shown in Figure 2A. Next, we explored the mechanism of how CDG activates the TLR4 pathway. Similar to the LPS challenge, CDG induced the formation of the TLR4/MD2 complex within 10 min, and this interaction could last for 15 min in WT MPMs (Figure 2B). Co‐immunoprecipitation (Co‐IP) studies further indicated that CDG increased the binding of MyD88 and TRIF with TLR4, but this binding could be reversed by blocking MD2 using an MD2‐neutralizing antibody (Figure 2C). The phosphorylated MAPKs (ERK, JNK, and p38) decreased in CDG‐stimulated MD2KO MPMs in comparison to CDG‐stimulated WT MPMs (Figure 2D, F, and G). Also, the levels of IκB‐α were elevated, whereas the phosphorylation of TBK1 and IRF3 were markedly descended in MD2KO MPMs when compared with the WT MPMs (Figure 2E, G, and H). To furthermore determine the main pathway in the CDG‐induced ALI, we constructed MyD88 and TRIF siRNA to knock down the expression of MyD88 and TRIF (siMyD88 and siTRIF) in MPMs, respectively. As shown in Supporting information Figure S1, siTRIF did not influence CDG‐induced activation of the MyD88 signalling pathway. Similarly, siMyD88 did not affect CDG‐induced activation of the TRIF signalling pathway either (Supporting information Figure S1). These findings indicate that MyD88‐ and TRIF‐dependent branches operate independently in CDG‐induced ALI. Besides, CDG elevated the expression of adhesion molecules such as ICAM1 and VCAM1 in WT MPMs, which was markedly reduced in CDG‐treated MD2KO MPMs (Figure 2I and J). We further confirmed these findings using a specific MD2 inhibitor, L6H21, in CDG‐treated WT MPMs. As shown in Supporting information Figure S2, L6H21 reduced the mRNA levels of CDG‐induced inflammatory cytokines (Supporting information Figure S2A–E), suppressed the CDG‐caused activation of MyD88‐ and TRIF‐dependent TLR4 cascades (Supporting information Figure S2F–I), and decreased the CDG‐induced adhesion molecule expression in WT MPMs (Supporting information Figure S2J–K). These findings indicate that CDG induced inflammation by inducing TLR4/MD2 complex formation and further activated both MyD88‐ and TRIF‐dependent cascades, suggesting that CDG mainly affects the activity of MD2 in the TLR4 pathway.

### 3.2 CDG interacts directly with MD2

**FIGURE 3:**
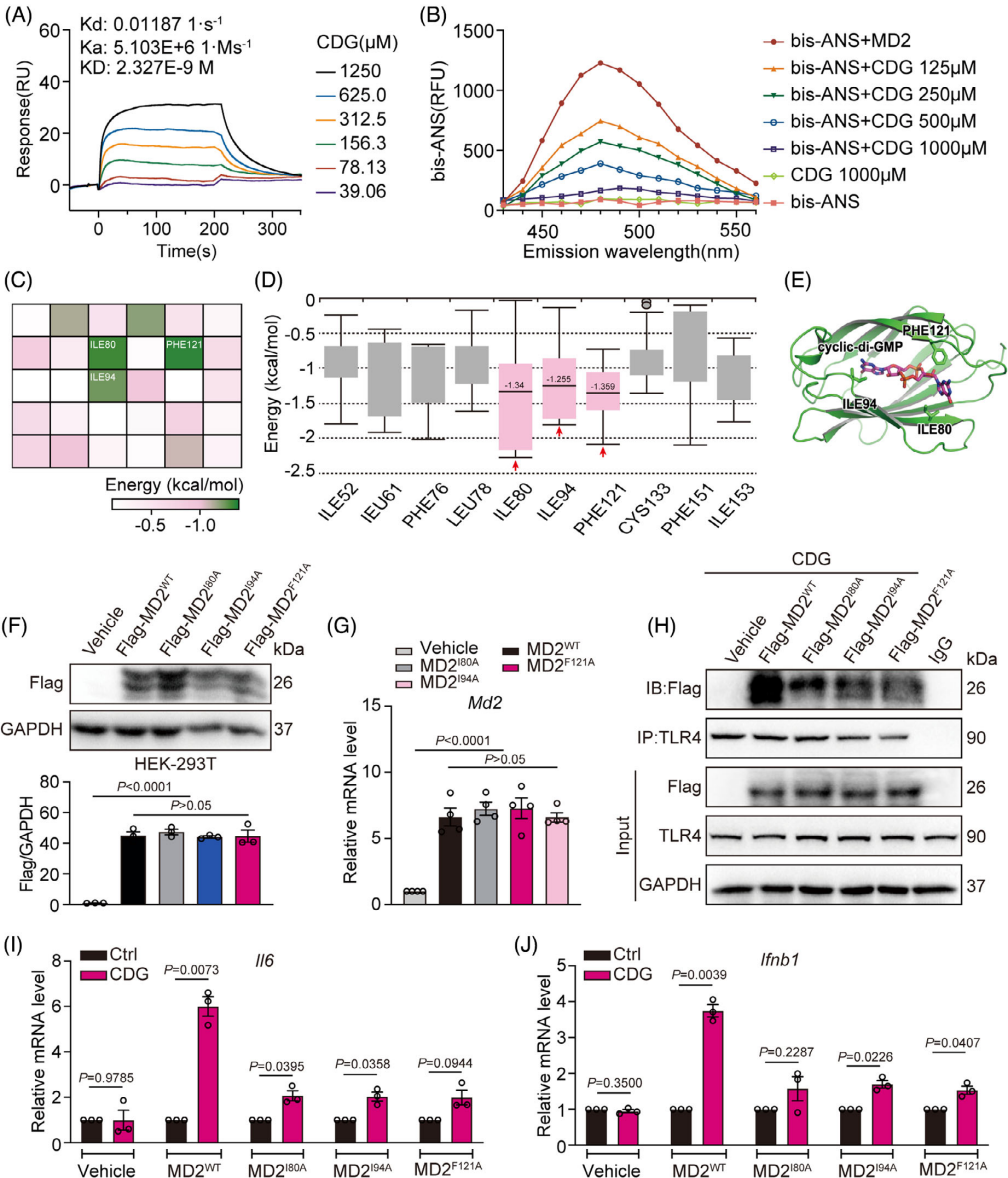
CDG interacts directly with MD2. (A) Surface plasmon resonance (SPR) analysis between CDG and rMD2. (B) The effects of CDG on the binding of fluorescent Bis‐ANS (5 µM) to rMD2. (C) Heatmap of average binding free energies for the top 30 residues in CDG‐MD2 molecular docking results. (D) Box plot of the per‐residue decomposition energy. € Molecular docking of CDG with MD2 protein was carried out with the programme Tripos molecular modelling packages Sybyl‐x.v1.1.083. (F, G) The overexpression efficiency of MD2WT and MD2Mut (I80A/I94A/F121A) plasmids in HEK‐293T cells and MD2KO‐derived MPMs was determined using Western blot assay (F) and RT‐qPCR assay (G). GAPDH was used as the loading control (*n* = 3 in each group, biological replicates). Data were normalized to the levels of *Actb* (*n* = 4 in each group, biological replicates). (H) HEK‐293T cells were stimulated with CDG (80 µM) for 15 min, and the effects of empty vehicle (Vehicle), MD2WT (MD2^WT^), or MD2Mut (MD2^I80A^, MD2^I94A^, MD2^F121A^) plasmids on MD2/TLR4 complex formation were assessed using co‐immunoprecipitation. (I, J) MD2KO‐derived MPMs were transfected with empty vehicle (Vehicle), MD2WT (MD2^WT^), or MD2Mut (MD2^I80A^, MD2^I94A^, MD2^F121A^) plasmids, respectively. Then the cells were exposed to CDG (80 µM) for 12 h. The mRNA levels of *Il6* (I) and *Ifnb1* (J) were measured via RT‐qPCR assay. Data were normalized to the levels of *Actb* (*n* = 3 in each group, biological replicates). Data information: In (F, G), data are presented as mean ± SEM, one‐way ANOVA followed by Dunnett's multiple comparisons test. In (I, J), data are presented as mean ± SEM, Student's *t*‐test.

It is well‐known that the direct binding of LPS and MD2 triggers the dimerization of TLR4/MD2 complexes, leading to the recruitment of both MyD88 and TRIF, ultimately activating intracellular signalling pathways.^28^, ^29^ Since CDG exhibited similar inflammatory effects to LPS, we speculated that CDG might directly target MD2 as well. Therefore, we investigated the physical interaction between CDG and MD2 using both surface plasmon resonance (SPR) and Bis‐ANS assays (Figure 3A and B). The SPR results revealed that CDG could directly bind to MD2 (KD value = 2.327 × 10^−9^ M). Similarly, Bis‐ANS fluorescence displacement assays showed reduced fluorescence signals after the treatment of CDG in a dosage‐dependent manner (Figure 3B). Interestingly, our observations indicated the binding affinity of CDG to either TLR4 or TLR2 was much lower when compared with the binding between CDG and MD2 even at the CDG concentration up to 1500 µM (Supporting information Figure S3), indicating the significance of MD2 in CDG‐mediating TLR4 signalling activation. All these results implicate that CDG is able to directly bind to MD2.

Next, we substantiated the binding sites of MD2 with CDG using computational docking and molecular simulation. As shown in Figure 3C–E and Supporting information Figure S4, isoleucine at positions 80 and 94, and phenylalanine at position 121 are the potential residues that interact with CDG, because they exhibit the lowest average binding energies, which are −1.340, −1.255, and −1.359 kcal/mol, respectively. Therefore, we mutated these three active amino acids of MD2 into alanine (MD2^WT^: wild type MD2; mutated MD2: MD2^I80A^, MD2^I94A^, MD2^F121A^). MD2 was overexpressed by transfecting MD2^WT^ and mutated MD2 plasmids in HEK‐293T cells (Figure 3F and G). Our data showed that CDG significantly induced MD2^WT^ binding to TLR4 (Figure 3H). However, triple‐mutated MD2 significantly reduced the binding affinity for CDG, indicating that isoleucine at positions 80 and 94, and phenylalanine at position 121 play vital roles in CDG‐MD2 binding (Figure 3H). Further investigation revealed that MD2KO MPMs, upon transfection with MD2^WT^ plasmid, regained their responsiveness to CDG (Figure 3I and J). However, the transcription levels of *Il6* and *Ifnb1* were reduced in MD2KO MPMs transfected with the mutated MD2 plasmids, revealing a decreased binding affinity between CDG and mutated MD2 (Figure 3I and J). These results are consistent with co‐IP assays, strengthening our conclusion that isoleucine at positions 80 and 94, and phenylalanine at position 121 are critical residues contributing to the interaction between MD2 and CDG.

### 3.3 MD2 plays a vital role in CDG‐induced inflammation in a STING‐independent manner

**FIGURE 4:**
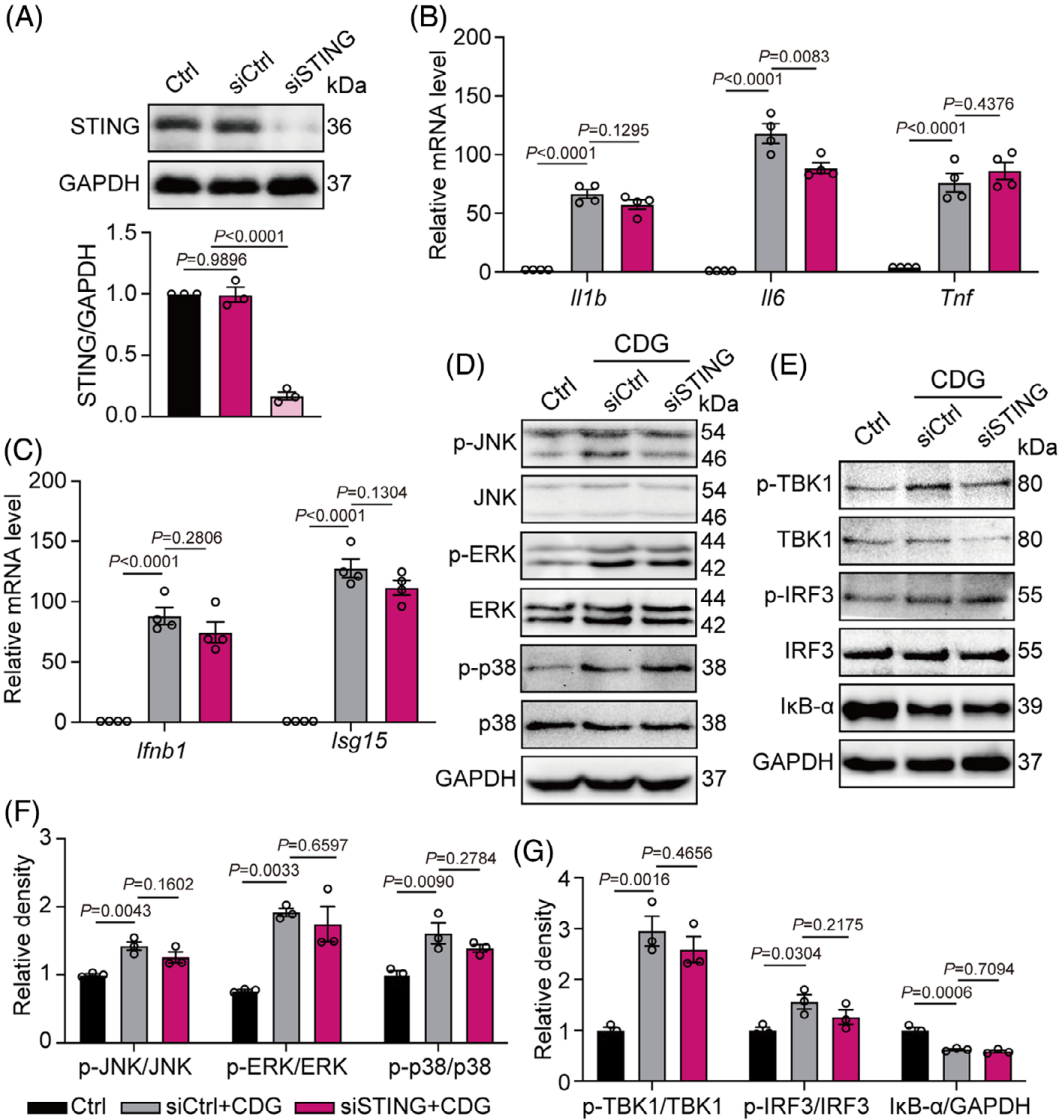
MD2 plays a vital role in CDG‐induced inflammation via a STING‐independent pathway. (A) MPMs were derived from WT mice. Immunoblot showing STING levels (*n* = 3 in each group, biological replicates) in MPMs following siRNA transfection [siSTING = STING siRNA, siCtrl = Ctrl siRNA]. (B, C) MPMs were derived from WT mice. STING‐silencing MPMs were stimulated with CDG (80 µM) for 12 h. mRNA levels of *Il1b*, *Il6*, *Tnf*, *Ifnb1*, and *Isg15* were measured via RT‐qPCR assay. Data were normalized to the levels of *Actb* (*n* = 4 in each group, biological replicates). (D–G) MPMs were derived from WT mice. STING‐silencing MPMs were stimulated with CDG (80 µM) for 1 h. Protein levels of IκB‐α and MAPK (ERK1/2, JNK, and p38), TBK1, and IRF3 were determined by Western blot assay. Unphosphorylated proteins and/or GAPDH were used as the loading control (*n* = 3 in each group, biological replicates). Representative blots (D, E) and densitometric quantification are shown (F, G). Data information: Data are presented as mean ± SEM. One‐way ANOVA followed by Dunnett's multiple comparisons test.

A large body of literature indicates bacteria‐derived cyclic dinucleotides (CDNs), such as CDG, can directly activate the STING pathway, leading to increased phosphorylation of TBK1 and nuclear trafficking of IRF3 and NF‐κB.^17^, ^30^ It is noteworthy that previous reports show that STING serves as intracellular damage sensor (sensing of cytosolic DNA) during infection with intracellular pathogenic, but not involved in extracellular pathogens.^31^, ^32^ It remains unclear whether CDG stimulates innate immune response outside the cell in a STING‐dependent or STING‐independent manner. To address this issue, we constructed STING short interfering RNA to down‐regulate STING expression (siSTING). As shown in Figure 4A, siSTING transfection achieved more than 75% knock‐down efficiency in MPMs. Under CDG treatment, transfection of siSTING only led to a decreased mRNA level of *Il6* (Figure 4B–G). The inhibitory effect of siSTING was much lower than that of MD2 deficiency (Figure 1E, Figure 2D–H). Moreover, the knockdown of STING did not significantly affect the CDG‐induced increase in mRNA levels of *Il1b*, *Tnf*, *Ifnb1* and *Isg15*, protein levels of phosphorylated MAPKs (ERK, JNK, and p38), or the phosphorylation of TBK1 and IRF3 (*p* > 0.05; Figure 4E–G). Collectively, these findings suggest that under the stimulation of extracellular CDG, MD2 plays a vital role in CDG‐induced inflammatory responses in a STING‐independent manner.

### 3.4 MD2 deficiency prevents CDG‐induced lung injury via suppressing TLR4 signaling pathway in vivo

**FIGURE 5:**
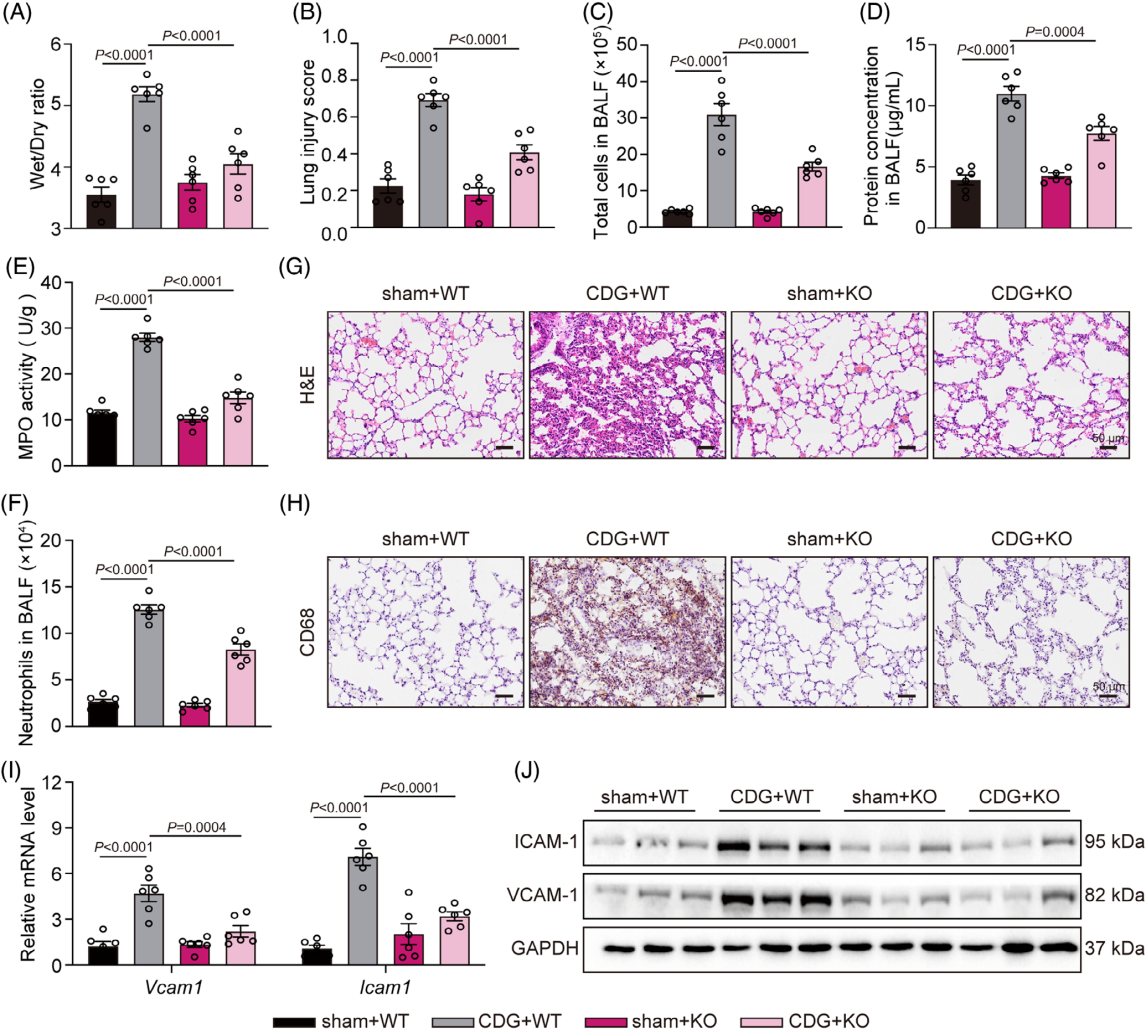
MD2 deficiency prevents CDG‐induced lung injury in vivo. (A) Lung wet/dry weight ratio (*n* = 6 in each group, biological replicates). (B) Quantification of the lung injury scores (*n* = 6 in each group, biological replicates). (C) Total cell counts in BALF samples were measured using a hemocytometer (*n* = 6 in each group, biological replicates). (D) Total protein concentration in BALF samples was measured (*n* = 6 in each group, biological replicates). (E) MPO activity levels in lung lysates (*n* = 6 in each group, biological replicates). (F) Neutrophils in BALF samples were assessed using Wright‐Giemsa staining (*n* = 6 in each group, biological replicates). (G) Representative H&E‐staining of lung tissues. Scale bar: 50 µm. (H) Immunohistochemical staining of lung tissues for CD68 macrophage markers. Scale bar: 50 µm. (I) mRNA levels of adhesion factors *Icam1* and *Vcam1* in lung tissues were measured via RT‐qPCR assay. Data were normalized to the levels of *Actb* (*n* = 6 in each group, biological replicates). (J) The protein levels of ICAM1 and VCAM1 were examined in lung tissues. GAPDH was used as the loading control. Data information: Data are presented as mean ± SEM. One‐way ANOVA followed by Dunnett's multiple comparisons test.

Previous studies involving intranasal administration of CDG, as a mucosal adjuvant, induced lung inflammation in mice.^33^, ^34^ Based on prior studies and the process of bacterial infection, we developed the ALI mouse model through intratracheal administration of CDG, similar to the LPS‐induced ALI mouse model.^4^ Global MD2 knockout (MD2KO) mice were confirmed using PCR (Supporting information Figure S5). Notably, MD2 deficiency significantly increased the survival rate of mice stimulated by CDG when compared with those of the CDG‐treated group (Supporting information Figure S6). As shown in Figure 5A and B, the CDG‐induced increase in wet‐to‐dry ratio and lung injury score was significantly alleviated in CDG‐treated MD2KO mice. Also, CDG stimulation increased total cell number (Figure 5C) and protein concentration (Figure 5D) in BALF, indicating abnormal permeability of the pulmonary vasculature. However, these changes were ameliorated in the MD2KO mice (Figure 5C and D). In addition, MPO activity in lung lysates and neutrophil counts in BALF samples were increased, while MD2 deficiency reversed these changes (Figure 5E and F). Immunohistochemistry staining is one of the current standard methods for estimating infiltrating immune cells, which is a sign of lung injury.^35^ H&E and immunohistochemistry staining assays showed that MD2 deficiency markedly inhibited CDG‐increased lung injury and immune cell infiltration (Figure 5G and H). Consistently, we found that CDG‐elevated expression of ICAM1 and VCAM1 both in the mRNA and protein levels, while MD2 deficiency significantly reduced the expression and transcription of the above adhesion molecules (Figure 5I and J). In summary, our findings suggest that MD2 deficiency prevents CDG‐induced lung injury in vivo.

**FIGURE 6:**
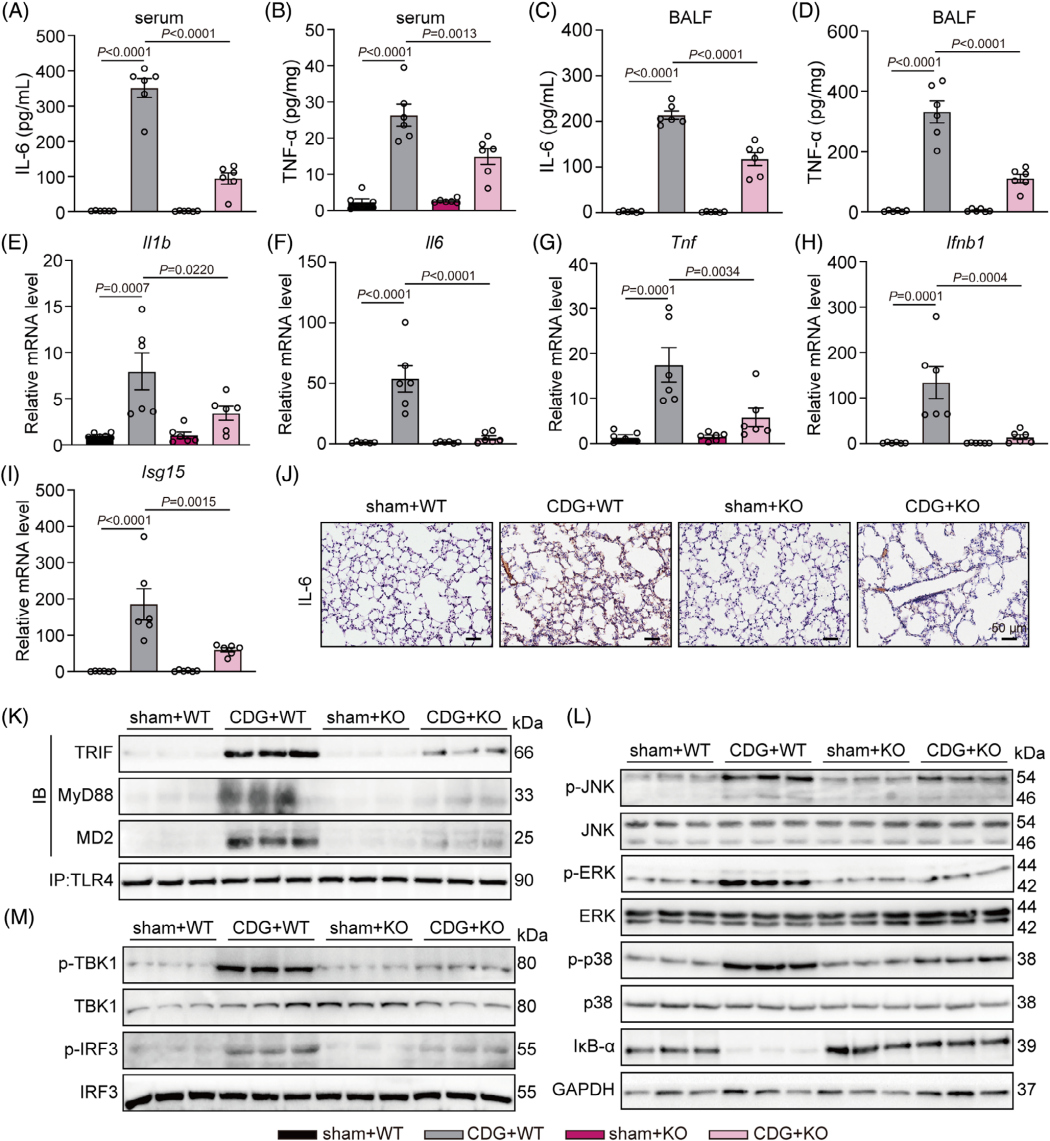
MD2 deficiency protects against CDG‐induced inflammation in vivo. (A–D) Levels of IL‐6 and TNF‐α in serum (A, B) and BALF (C, D) samples from mice challenged with CDG (*n* = 6 in each group, biological replicates). (E–I) mRNA levels of *Il1b*, *Il6*, *Tnf*, *Ifnb1*, and *Isg15* in lung tissues were measured via RT‐qPCR assay. Data were normalized to the levels of *Actb* (*n* = 6 in each group, biological replicates). (J) Representative immunostaining of mouse lung tissues for IL‐6. Scale bar: 50 µm. (K) MD2‐TLR4 pathway activation was assessed in lung lysates from mice. TLR4 was immunoprecipitated (IP), and the associated TRIF, MD2, and MyD88 levels were detected using immunoblotting (IB). (L–M) MD2‐TLR4 pathway activation was assessed in lung lysates from mice. Activation of the MyD88‐dependent (L) and TRIF‐dependent (M) pathways was assessed using Western blot assays. Unphosphorylated proteins and GAPDH were used as the loading controls. Data information: Data are presented as mean ± SEM. One‐way ANOVA followed by Dunnett's multiple comparisons test.

Next, we assessed the anti‐inflammatory effects of MD2 deficiency in the CDG‐induced ALI mouse model. As depicted in Figure 6A–D, CDG significantly upregulated IL‐6 and TNF‐α protein levels in both serum and BALF, while MD2 deficiency markedly reduced these upregulations. The RT‐qPCR results revealed that CDG increased inflammatory cytokines transcription in lung tissues, while MD2 deficiency significantly suppressed the up‐regulation (Figure 6E–I). In addition, similar results were found in the IL‐6 immunohistochemical staining in lung tissues (Figure 6J). Next, we investigated whether CDG exerts pro‐inflammatory activity by targeting MD2 in vivo. Co‐IP assays confirmed the interaction of TLR4 with MD2, TRIF, and MyD88 in lung tissues after CDG stimulation, which could be significantly suppressed in MD2KO mice (Figure 6K and Supporting information Figure S7A). As depicted in Figure 6M and L, Supporting information Figure S7B–D, CDG induced the activation of TRIF‐ and MyD88‐dependent pathways, which was markedly downregulated in MD2KO mice. All these data suggest that MD2 deficiency alleviates CDG‐induced inflammatory responses in ALI mice.

### 3.5 MD2 inhibitor suppresses CDG‐induced inflammatory lung injury in vivo

**FIGURE 7:**
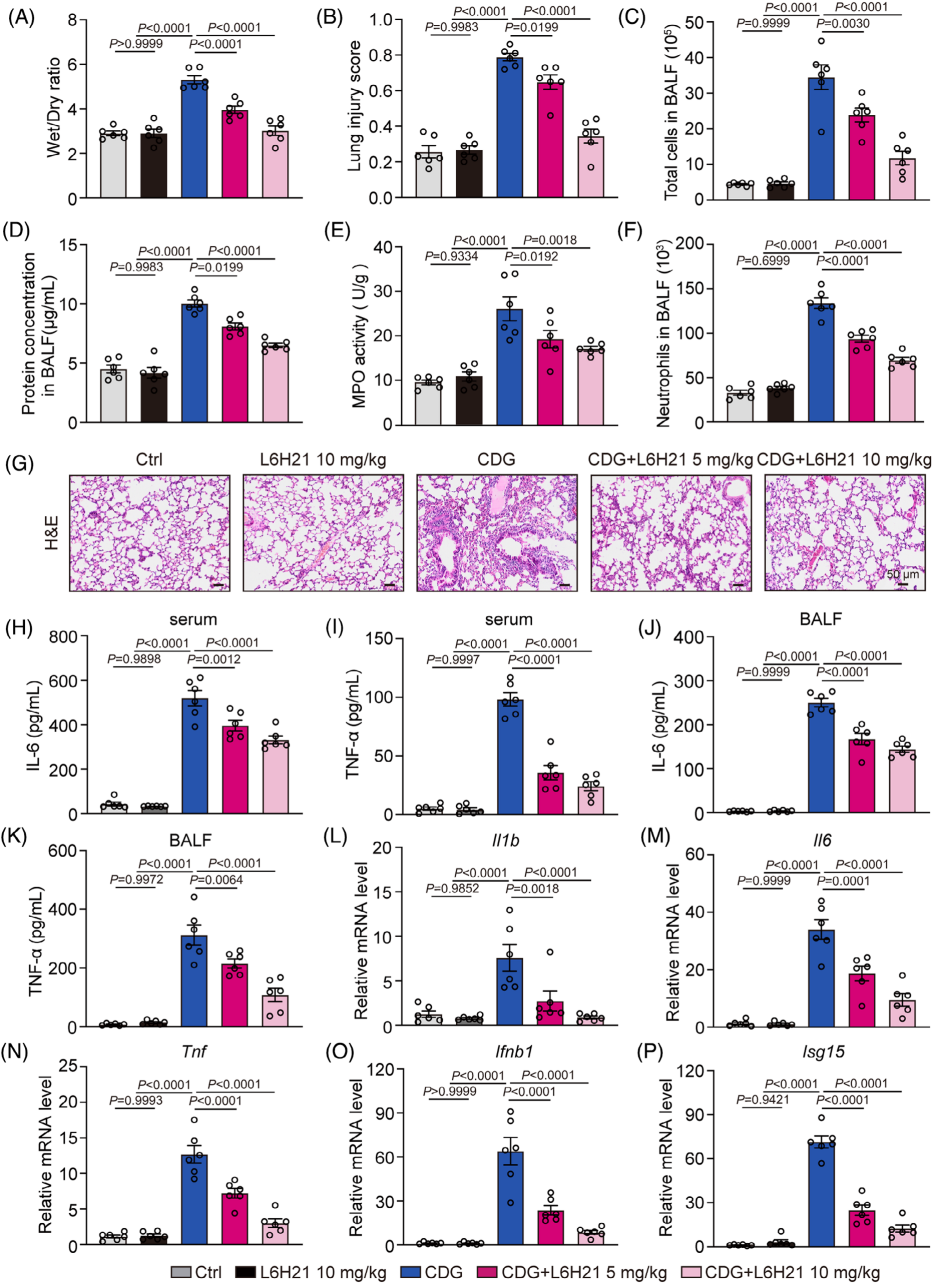
MD2 inhibitor suppresses CDG‐induced acute lung injury in vivo. (A) WT mice were intraperitoneally administered with L6H21 (5 or 10 mg/kg/12 h) for 36 h prior to the CDG challenge (3 mg/kg) for 6 h. Lung wet/dry weight ratio (*n* = 6 in each group, biological replicates). (B) Quantification of the lung injury scores (*n* = 6 in each group, biological replicates). (C) Total cell counts in BALF samples were measured using a hemocytometer (*n* = 6 in each group, biological replicates). (D) Total protein concentration in BALF samples was measured (*n* = 6 in each group, biological replicates). (E) MPO activity levels in lung lysates (*n* = 6 in each group, biological replicates). (F) Neutrophils in BALF samples were assessed using Wright‐Giemsa staining (*n* = 6 in each group, biological replicates). (G) Representative H&E staining of lung tissues. Scale bar: 50 µm. (H–K) Levels of IL‐6 and TNF‐α in serum (H, I) and BALF (J, K) samples from mice challenged with CDG (*n* = 6 in each group, biological replicates). (L–P) mRNA levels of *Il1b*, *Il6*, *Tnf*, *Ifnb1*, and *Isg15* in lung tissues were measured via RT‐qPCR assay. Data were normalized to the levels of *Actb* (*n* = 6 in each group, biological replicates). Data information: Data are presented as mean ± SEM. One‐way ANOVA followed by Dunnett's multiple comparisons test.

Furthermore, we evaluated the pharmacological effect of the MD2 inhibitor, L6H21, against CDG‐induced lung injury in vivo. Notably, L6H21 treatment significantly increased the survival rate of mice stimulated by CDG (Supporting information Figure S6). As shown in Figure 7A–F, the index of lung injury (wet/dry ratio, total cells and protein concentration and neutrophils in BALF, MPO activity and lung injury score) were significantly increased by CDG‐challenge, but significantly alleviated in CDG plus L6H21 treatment in a dose‐dependent manner. H&E staining revealed that L6H21 reduced CDG‐induced inflammatory cell infiltration and thickening of alveolar walls, ultimately mitigating damage to the lung tissue structure (Figure 7G). In addition, L6H21 treatment reversed CDG‐induced upregulation of IL‐6 and TNF‐α expression in both serum and BALF samples (Figure 7H–K). Further evaluation of pulmonary inflammation revealed a significant down‐regulation of *Il1b*, *Il6*, *Tnf*, *Ifnb1* and *Isg15* mRNA transcription after L6H21 treatment (Figure 7L–P). In summary, our data confirm that MD2 inhibitor effectively alleviates CDG‐induced ALI via inhibiting inflammation in mice.

## 4 DISCUSSION

In this study, we aim to verify how CDG stimulates innate immune response outside host cells and investigate its underlying molecular mechanisms. During infection, extracellular bacteria autolyze or rupture, releasing CDG to the host cell's vicinity.^36^, ^37^ Thus, we speculated that CDG might act as a PAMP molecule, capable of eliciting inflammatory responses via Toll‐like receptors or their accessory proteins. Our results provide strong support that MD2 is a crucial accessory protein in CDG‐induced proinflammatory activities and lung injury. The five key findings of this study are as follows: (1) intratracheal injection of CDG could mediate lung injury, which is a novel ALI animal model; (2) CDG could function without entering the cell in a STING‐independent manner; (3) CDG directly bound to MD2, inducing the activation of the TLR4 signalling pathway; (4) isoleucine at positions 80 and 94, and phenylalanine at position 121 of MD2 protein are the three primary residues involved in the interaction with CDG; (5) MD2 inhibitor suppressed the production of proinflammatory molecules in lung tissue, thereby attenuating CDG‐induced lung injury in vivo. Most importantly, our data not only reveal CDG as a novel PAMP molecule that interacts with MD2 but also prove that MD2 could be a therapeutic target for COVID‐19 patients with bacterial co‐infection.

It is noteworthy that previous reports prove that STING serves as an intracellular damage sensor (sensing of cytosolic DNA) during infection with intracellular pathogenic, but not involved in extracellular pathogens.^31^, ^32^ Recent years have developed inhibitors targeting cGAS‐STING‐TBK1 with benefits in the treatment of inflammation, virus infection, bacterial infection, and cancers.^38^, ^39^, ^40^ In a groundbreaking study, Ablasser et al.^41^ introduced a novel compound known as H‐151, which demonstrated its remarkable capability to mitigate systemic cytokine responses induced by STING agonists, effectively functioning as a potent STING inhibitor in vivo. Meanwhile, Li et al.^42^ demonstrated that astin C, isolated from the traditional Chinese medicinal plant *Aster tataricus*, inhibits cytosolic DNAs‐mediated STING signalling and innate inflammatory responses as a STING‐specific small‐molecular inhibitor. Thus, previous studies are mainly about the process of intracellular bacteria released‐CDG, which focuses on cytosolic DNAs‐mediated inflammatory responses. It has been more than ten years since McWhirter and his team first discovered that high concentrations of overlapping CDG can also induce IFN‐β.^19^ Wang et al.^22^ found extracellular CDG increased COX‐2 secretion in macrophages in a STING‐independent manner. Besides, one study indicated that incubation of human gingival fibroblasts with CDG led to a significant increase in IL‐8 production, while another study proved that incubation of human gingival keratinocytes (HMK cells) with CDG led to a significant increase in MCP‐1 expression.^36^, ^37^ To date, the mechanism how extracellular CDG is involved in mediating inflammatory responses is still unknown. Our study fills the knowledge gap by proving the mechanism of how CDG directly binds to MD2 and activates the TLR4 signalling.

Many studies have reported that MD2 deficiency restrained LPS‐induced ALI, and therapeutic drugs and methods targeting MD2 have effective therapeutic effects in the treatment of ALI/ARDS, such as natural compounds Licochalcone A and Chalcone.^4^, ^43^, ^44^ Therefore, MD2 has been shown to be an effective target for ALI/ARDS. Our paper not only enriches the molecular mechanisms of extracellular CDG but also expands the scope of the application of MD2 as a therapeutic target for treating lung injury diseases associated with ALI/ARDS. However, there are some limitations in our study. Because the overall responses of host cells to pathogens are an aggregate of multiple stimuli, it is still uncertain which plays a more important role in bacterial infection: the extracellular CDG or the intracellular CDG. Indeed, our data show that MD2 deficiency could not completely reverse CDG‐induced lung injury (Figures 5, 6 and Supporting information Figure S6), suggesting the potential of other mediators involved in CDG activity. Besides MD2, both STING and DDX41 have been identified to directly bind to CDG.^45^, ^46^ It is worth noting that both STING and DDX41 are located exclusively in the cytoplasm. It is possible that part of the CDG, which enters the host cells through efflux pumps or phagocytosis, might also participate in the pathology of CDG‐induced ALI. Thus, further studies are needed to figure out whether extracellular or intracellular CDG could induce stronger inflammatory responses during bacterial infection. For example, a model of direct infection with bacterial strains (e.g., *Pseudomonas aeruginosa*, *Klebsiella pneumoniae*, and *Staphylococcus aureus*) in host cells or animal models is needed to mimic the clinical infection process. In addition, based on our current data, the ability of LPS to activate innate immune responses seems stronger than that of CDG (Figure 1C and D). It would be difficult to compare the proinflammatory effect of LPS and CDG in patients since it could be affected by the types, toxicity and infection modes of bacteria. Another limitation of our study is that we did not demonstrate the expression of MD2 in human COVID‐19 lung tissues or the expression of the TLR4/MD2 complex in peripheral blood mononuclear cells from COVID‐19‐positive individuals due to technical and resource constraints to obtain these specific human samples in our lab. Nonetheless, our study provides compelling evidence demonstrating the involvement of the macrophage membrane recognition receptor, MD2, in CDG‐induced lung injury and inflammation, hinting at CDG as a novel PAMP potentially implicated in bacterial infection‐induced lung inflammation, which reveals a potential therapeutic strategy in the clinic.

Bacterial co‐infection is a relatively common complication of COVID‐19 and is diagnosed in 9.1% (102/1125) of hospitalized adults^47^ as well as 40% (8/20) of pediatric patients.^48^ Recent research reveals a synergistic effect between the SARS‐CoV‐2 S protein and LPS, highlighting that individuals with ALI or ARDS are susceptible to severe COVID‐19 complications.^49^ Therefore, our study might suggest that CDG potentially plays a role in the course of COVID‐19. Interestingly, Zhao et al.^50^ implicated that trimeric spike proteins from SARS‐CoV‐2 or SARS‐CoV could elicit an anti‐bacterial‐like response at the early stage of infection through TLR4 or TLR4‐related signalling pathways. Meanwhile, Zhao et al.^50^ further proved that MD2 was involved in spike protein‐mediated TLR4 activation. Thus, according to the current study, the TLR4/MD2 complex could be a new therapeutic target in bacterial co‐infections. Further validation is required regarding the direct role of CDG in COVID‐19, which presents as one of our future research directions.

## 5 CONCLUSION

In conclusion, our paper provides evidence of CDG mediating lung injury through direct interaction with MD2. Our findings indicate that MD2 blockade using a specific inhibitor or genetic knockout is able to attenuate CDG‐induced ALI by reducing TLR4‐mediated inflammation. Isoleucine at positions 80 and 94, and phenylalanine at position 121 are the critical residues in the binding of MD2 to CDG. This finding not only enriches the mechanisms of bacterial infection but also brings new perspectives to the treatment of bacterial infectious diseases. Additionally, this new finding of the mechanism may lead to new treatment strategies for bacterial co‐infection in patients with COVID‐19.

## AUTHOR CONTRIBUTIONS

Chenchen Qian, Yi Wang, and Guang Liang contributed to the literature search and study design. Chenchen Qian, Weiwei Zhu, Jiong Wang, Weiyang Tang, Xin Liu, Yuyang Zhang, and Yong Xu performed the experiments and analyzed the data. Zhe Wang, Bo Jin, and Xin Liu provided the technologies and the methods. Chenchen Qian, Guang Liang, and Yi Wang participated in the drafting of the article. All authors reviewed and approved the manuscript.

## CONFLICT OF INTEREST STATEMENT

The authors declare no conflict of interest.

## ETHICS STATEMENT

Ethical approval for all animal care and experimental procedures was obtained from the Animal Policy and Welfare Committee of Wenzhou Medical University (approval number: wydw2022‐0312).

## Supporting information

Supporting Information

## Data Availability

The data that support the findings of this study are available from the corresponding author upon reasonable request. Some data may not be made available because of privacy or ethical restrictions.

## References

[1] He Y‐Q , Zhou C‐C , Yu L‐Y , et al. Natural product derived phytochemicals in managing acute lung injury by multiple mechanisms. Pharmacol Res. 2021;163:105224. 33007416 10.1016/j.phrs.2020.105224 PMC7522693

[2] Liu C , Xiao K , Xie L . Advances in the use of exosomes for the treatment of ALI/ARDS. Front Immunol. 2022;13:971189. 36016948 10.3389/fimmu.2022.971189 PMC9396740

[3] Fan EKY , Fan J . Regulation of alveolar macrophage death in acute lung inflammation. Respir Res. 2018;19(1):50. 29587748 10.1186/s12931-018-0756-5 PMC5872399

[4] Zhu W , Wang M , Jin L , et al. Licochalcone A protects against LPS‐induced inflammation and acute lung injury by directly binding with myeloid differentiation factor 2 (MD2). Br J Pharmacol. 2023;180(8):1114‐1131. 36480410 10.1111/bph.15999

[5] Collaborators G . Global mortality associated with 33 bacterial pathogens in 2019: a systematic analysis for the Global Burden of Disease Study 2019. Lancet. 2022;400(10369):2221‐2248. 36423648 10.1016/S0140-6736(22)02185-7 PMC9763654

[6] Vidaur L , Eguibar I , Olazabal A , et al. Impact of antimicrobial stewardship in organisms causing nosocomial infection among COVID‐19 critically ill adults. Eur J Intern Med. 2024;119:93‐98. 37580243 10.1016/j.ejim.2023.08.009

[7] Gan Y , Zhang G , Sun H , Lyu X . Clinical characteristics and risk factors for bacterial co‐infections in COVID‐19 patients: a retrospective study. J Glob Antimicrob Resist. 2024. 10.1016/j.jgar.2024.04.007 38723711

[8] Naqvi I , Giroux N , Olson L , et al. DAMPs/PAMPs induce monocytic TLR activation and tolerance in COVID‐19 patients; nucleic acid binding scavengers can counteract such TLR agonists. Biomaterials. 2022;283:121393. 35349874 10.1016/j.biomaterials.2022.121393 PMC8797062

[9] Schröder NWJ , Morath S , Alexander C , et al. Lipoteichoic acid (LTA) of *Streptococcus pneumoniae* and *Staphylococcus aureus* activates immune cells via Toll‐like receptor (TLR)‐2, lipopolysaccharide‐binding protein (LBP), and CD14, whereas TLR‐4 and MD‐2 are not involved. J Biol Chem. 2003;278(18):15587‐15594. 12594207 10.1074/jbc.M212829200

[10] Bitto NJ , Cheng L , Johnston EL , et al. Staphylococcus aureus membrane vesicles contain immunostimulatory DNA, RNA and peptidoglycan that activate innate immune receptors and induce autophagy. J Extracell Vesicles. 2021;10(6):e12080. 33815695 10.1002/jev2.12080 PMC8015888

[11] Wang F , Li Y , Yang C , et al. Mannan‐binding lectin suppresses peptidoglycan‐induced TLR2 activation and inflammatory responses. Mediators Inflamm. 2019;2019:1349784. 30728747 10.1155/2019/1349784 PMC6343158

[12] Akahoshi DT , Bevins CL . Flagella at the host‐microbe interface: key functions intersect with redundant responses. Front Immunol. 2022;13:828758. 35401545 10.3389/fimmu.2022.828758 PMC8987104

[13] Almblad H , Randall TE , Liu F , et al. Bacterial cyclic diguanylate signaling networks sense temperature. Nat Commun. 2021;12(1):1986. 33790266 10.1038/s41467-021-22176-2 PMC8012707

[14] de Moura Rodrigues D , Lacerda‐Queiroz N , Couillin I , Riteau N . STING targeting in lung diseases. Cells. 2022;11(21). 10.3390/cells11213483 PMC9657237 36359882

[15] Couillin I , Riteau N . STING signaling and sterile inflammation. Front Immunol. 2021;12:753789. 34659260 10.3389/fimmu.2021.753789 PMC8517477

[16] Parvatiyar K , Zhang Z , Teles RM , et al. The helicase DDX41 recognizes the bacterial secondary messengers cyclic di‐GMP and cyclic di‐AMP to activate a type I interferon immune response. Nat Immunol. 2012;13(12):1155‐1161. 23142775 10.1038/ni.2460 PMC3501571

[17] Oduro PK , Zheng X , Wei J , et al. The cGAS‐STING signaling in cardiovascular and metabolic diseases: future novel target option for pharmacotherapy. Acta Pharm Sin B. 2022;12(1):50‐75. 35127372 10.1016/j.apsb.2021.05.011 PMC8799861

[18] Tian X , Liu C , Wang Z . The induction of inflammation by the cGAS‐STING pathway in human dental pulp cells: a laboratory investigation. Int Endod J. 2022;55(1):54‐63. 34570917 10.1111/iej.13636

[19] Mcwhirter SM , Barbalat R , Monroe KM , et al. A host type I interferon response is induced by cytosolic sensing of the bacterial second messenger cyclic‐di‐GMP. J Exp Med. 2009;206(9):1899‐1911. 19652017 10.1084/jem.20082874 PMC2737161

[20] Kumagai Y , Matsuo J , Hayakawa Y , Rikihisa Y . Cyclic di‐GMP signaling regulates invasion by *Ehrlichia chaffeensis* of human monocytes. J Bacteriol. 2010;192(16):4122‐4133. 20562302 10.1128/JB.00132-10 PMC2916414

[21] Sooreshjani MA , Gursoy UK , Aryal UK , Sintim HO . Proteomic analysis of RAW macrophages treated with cGAMP or c‐di‐GMP reveals differentially activated cellular pathways. RSC Adv. 2018;8(64):36840‐36851. 35558957 10.1039/c8ra04603d PMC9089301

[22] Wang M , Chaudhuri R , Ong WWS , Sintim HO . c‐di‐GMP induces COX‐2 expression in macrophages in a STING‐independent manner. ACS Chem Biol. 2021;16(9):1663‐1670. 34478263 10.1021/acschembio.1c00342

[23] Wu J , Li J , Cai Y , et al. Evaluation and discovery of novel synthetic chalcone derivatives as anti‐inflammatory agents. J Med Chem. 2011;54(23):8110‐8123. 21988173 10.1021/jm200946h

[24] Matute‐Bello G , Downey G , Moore BB , et al. An official American Thoracic Society workshop report: features and measurements of experimental acute lung injury in animals. Am J Respir Cell Mol Biol. 2011;44(5):725‐738. 21531958 10.1165/rcmb.2009-0210ST PMC7328339

[25] Huang X , Xiu H , Zhang S , Zhang G . The role of macrophages in the pathogenesis of ALI/ARDS. Mediators Inflamm. 2018;2018:1264913. 29950923 10.1155/2018/1264913 PMC5989173

[26] Wu Y , Yu X , Wang Y , et al. Ruscogenin alleviates LPS‐triggered pulmonary endothelial barrier dysfunction through targeting NMMHC IIA to modulate TLR4 signaling. Acta Pharm Sin B. 2022;12(3):1198‐1212. 35530141 10.1016/j.apsb.2021.09.017 PMC9069402

[27] Zhao R , Wang L , Wang T , Xian P , Wang H , Long Q . Inhalation of MSC‐EVs is a noninvasive strategy for ameliorating acute lung injury. J Control Release. 2022;345:214‐230. 35307508 10.1016/j.jconrel.2022.03.025

[28] Wang Y , Su L , Morin MD , et al. TLR4/MD‐2 activation by a synthetic agonist with no similarity to LPS. Proc Natl Acad Sci USA. 2016;113(7):E884‐893. 26831104 10.1073/pnas.1525639113 PMC4763747

[29] Rajaiah R , Perkins DJ , Ireland DDC , Vogel SN . CD14 dependence of TLR4 endocytosis and TRIF signaling displays ligand specificity and is dissociable in endotoxin tolerance. Proc Natl Acad Sci USA. 2015;112(27):8391‐8396. 26106158 10.1073/pnas.1424980112 PMC4500272

[30] Wu J‐J , Zhao L , Hu H‐G , Li W‐H , Li Y‐M . Agonists and inhibitors of the STING pathway: potential agents for immunotherapy. Med Res Rev. 2020;40(3):1117‐1141. 31793026 10.1002/med.21649

[31] Dejmek M , Šála M , Brazdova A , et al. Discovery of isonucleotidic CDNs as potent STING agonists with immunomodulatory potential. Structure. 2022;30(8):1146‐1156. e11. 35690061 10.1016/j.str.2022.05.012

[32] Hu Q , Zhou Q , Xia X , et al. Cytosolic sensor STING in mucosal immunity: a master regulator of gut inflammation and carcinogenesis. J Exp Clin Cancer Res. 2021;40(1):39. 33485379 10.1186/s13046-021-01850-9 PMC7825222

[33] Subramanian H , Hashem T , Bahal D , Kammala AK , Thaxton K , Das R . Ruxolitinib ameliorates airway hyperresponsiveness and lung inflammation in a corticosteroid‐resistant murine model of severe asthma. Front Immunol. 2021;12:786238. 34777398 10.3389/fimmu.2021.786238 PMC8586657

[34] Cavagnero KJ , Badrani JH , et al. Cyclic‐di‐GMP induces STING‐dependent ILC2 to ILC1 shift during innate type 2 lung inflammation. Front Immunol. 2021;12:618807. 33679760 10.3389/fimmu.2021.618807 PMC7935536

[35] Nguyen N , Xu S , Lam TYW , Liao W , Wong WSF , Ge R . ISM1 suppresses LPS‐induced acute lung injury and post‐injury lung fibrosis in mice. Mol Med. 2022;28(1):72. 35752760 10.1186/s10020-022-00500-w PMC9233842

[36] Elmanfi S , Zhou J , Sintim HO , Könönen E , Gürsoy M , Gürsoy UK . Regulation of gingival epithelial cytokine response by bacterial cyclic dinucleotides. J Oral Microbiol. 2019;11(1):1538927. 30598733 10.1080/20002297.2018.1538927 PMC6263105

[37] Elmanfi S , Sintim HO , Zhou J , Gürsoy M , Könönen E , Gürsoy UK . Activation of gingival fibroblasts by bacterial cyclic dinucleotides and lipopolysaccharide. Pathogens. 2020;9(10). 10.3390/pathogens9100792 PMC7600373 32993127

[38] Ding C , Song Z , Shen A , Chen T , Zhang Ao . Small molecules targeting the innate immune cGAS‒STING‒TBK1 signaling pathway. Acta Pharm Sin B. 2020;10(12):2272‐2298. 33354501 10.1016/j.apsb.2020.03.001 PMC7745059

[39] Christensen MH , Jensen SB , Miettinen JJ , et al. HSV‐1 ICP27 targets the TBK1‐activated STING signalsome to inhibit virus‐induced type I IFN expression. EMBO J. 2016;35(13):1385‐1399. 27234299 10.15252/embj.201593458 PMC4931188

[40] Chen Y‐A , Shen Y‐L , Hsia H‐Y , Tiang Y‐P , Sung T‐L , Chen L‐Y . Extrachromosomal telomere repeat DNA is linked to ALT development via cGAS‐STING DNA sensing pathway. Nat Struct Mol Biol. 2017;24(12):1124‐1131. 29106411 10.1038/nsmb.3498

[41] Haag SM , Gulen MF , Reymond L , et al. Targeting STING with covalent small‐molecule inhibitors. Nature. 2018;559(7713):269‐273. 29973723 10.1038/s41586-018-0287-8

[42] Li S , Hong Ze , Wang Z , et al. The cyclopeptide Astin C specifically inhibits the innate immune CDN sensor STING. Cell Rep. 2018;25(12):3405‐3421. e7. 30566866 10.1016/j.celrep.2018.11.097

[43] Luo Wu , Yang Li‐B , Qian C‐C , et al. Flavokawain B alleviates LPS‐induced acute lung injury via targeting myeloid differentiation factor 2. Acta Pharmacol Sin. 2022;43(7):1758‐1768. 34737421 10.1038/s41401-021-00792-4 PMC9253132

[44] Zhang Ya‐Li , Zhang W‐X , Yan J‐Q , et al. Chalcone derivatives ameliorate lipopolysaccharide‐induced acute lung injury and inflammation by targeting MD2. Acta Pharmacol Sin. 2022;43(1):76‐85. 34480112 10.1038/s41401-021-00764-8 PMC8724327

[45] Burdette DL , Monroe KM , Sotelo‐Troha K , et al. STING is a direct innate immune sensor of cyclic di‐GMP. Nature. 2011;478(7370):515‐518. 21947006 10.1038/nature10429 PMC3203314

[46] Jiang Y , Zhu Y , Liu Z‐J , Ouyang S . The emerging roles of the DDX41 protein in immunity and diseases. Protein Cell. 2017;8(2):83‐89. 27502187 10.1007/s13238-016-0303-4 PMC5291771

[47] Moreno‐García E , Puerta‐Alcalde P , Letona L , et al. Bacterial co‐infection at hospital admission in patients with COVID‐19. Int J Infect Dis. 2022;118:197‐202. 35257905 10.1016/j.ijid.2022.03.003 PMC8896874

[48] Xia W , Shao J , Guo Yu , Peng X , Li Z , Hu D . Clinical and CT features in pediatric patients with COVID‐19 infection: different points from adults. Pediatr Pulmonol. 2020;55(5):1169‐1174. 32134205 10.1002/ppul.24718 PMC7168071

[49] Petruk G , Puthia M , Petrlova J , et al. SARS‐CoV‐2 spike protein binds to bacterial lipopolysaccharide and boosts proinflammatory activity. J Mol Cell Biol. 2020;12(12):916‐932. 33295606 10.1093/jmcb/mjaa067 PMC7799037

[50] Zhao Y , Kuang M , Li J , et al. SARS‐CoV‐2 spike protein interacts with and activates TLR41. Cell Res. 2021;31(7):818‐820. 33742149 10.1038/s41422-021-00495-9 PMC7975240

