# Supplementary material for: Cyclic‐di‐GMP induces inflammation and acute lung injury through direct binding to MD2
Source: Clin Transl Med. 2024 Aug 21;14(8):e1744. doi: 10.1002/ctm2.1744 (PMC11337466; doi:10.1002/ctm2.1744)
Supplement: Supplementary file 1 — Supporting Information [file CTM2-14-e1744-s001.docx]

Supporting Information

**Cyclic-di-GMP induces inflammation and lung injury through direct binding to MD2**

This supplementary file includes 2 supporting tables and 6 supporting figures.

1. **Supporting tables**

**Table S1.** Lung injury score evaluation system

| **Parameter** | **Score per field** | | |
| --- | --- | --- | --- |
|  | **0** | **1** | **2** |
|  | 0 | 1-5 | ＞5 |
| *b. Neutrophils in the interstitial space* | 0 | 1-5 | ＞5 |
| *c. Hyaline membranes* | 0 | 1 | ＞1 |
| *d. Proteinaceous debris filling the airspaces* | 0 | 1 | ＞1 |
| *e. Alveolar septal thickening* | ＜2× | 2×-4× | ＞4× |

**Table S2.** Sequences of primers used for real-time qPCR assay

| **Gene** | **Species** | **Forward** | **Reverse** |
| --- | --- | --- | --- |
| *Tlr4* | Mouse | AGCTTCTCCAATTTTTCAGAACTTC | TGAGAGGTGGTGTAAGCCATGC |
| *Md2* | Mouse | CTGAACCCTGCATAAGACTGAGG | CTTCCTTACGCTTCGGCAACTC |
| *Icam1* | Mouse | GCCTTGGTAGAGGTGACTGAG | GACCGGAGCTGAAAAGTTGTA |
| *Vcam1* | Mouse | TGCCGAGCTAAATTACACATTG | CCTTGTGGAGGGATGTACAGA |
| *Il1β* | Mouse | TCGCAGCAGCACATCAACAAGAG | AGGTCCACGGGAAAGACACAGG |
| *Il6* | Mouse | CTCCCAACAGACCTGTCTATAC | CCATTGCACAACTCTTTTCTCA |
| *Isg15* | Mouse | GGTGTCCGTGACTAACTCCAT | TGGAAAGGGTAAGACCGTCCT |
| *Ifnb1* | Mouse | CAGCTCCAAGAAAGGACGAAC | GGCAGTGTAACTCTTCTGCAT |
| *Tnf* | Mouse | ATGTCTCAGCCTCTTCTCATTC | GCTTGTCACTCGAATTTTGAGA |
| *Actb* | Mouse | CTACCTCATGAAGATCCTGACC | CACAGCTTCTCTTTGATGTCAC |

1. **Supporting figures**

**
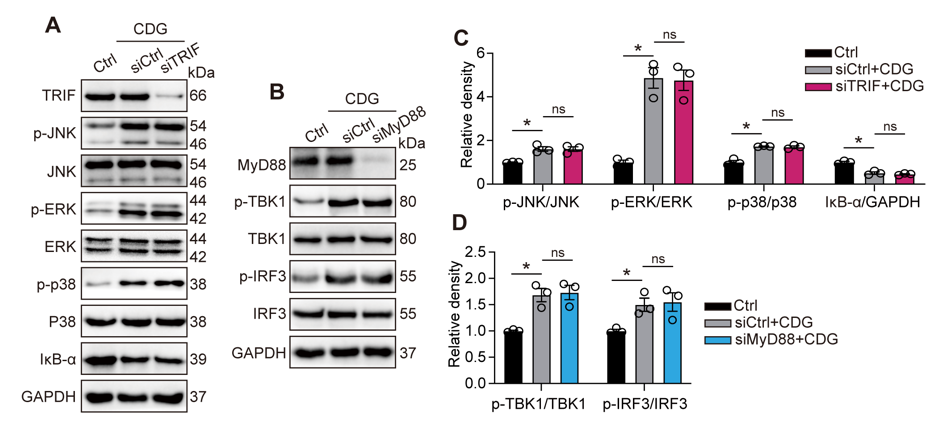
**

**Supplementary Figure S1.** **MyD88- and TRIF-mediated pathways play independent roles in CDG-induced inflammation.** MPMs were derived from C57BL/6J mice. TRIF or MyD88-silencing MPMs were stimulated with CDG (80 μM) for 1 hour. Protein levels of IκB-α and MAPK (ERK1/2, JNK, and p38), TBK1, and IRF3 were determined by Western blot. Unphosphorylated proteins and/or GAPDH were used as the loading controls (n = 3 in each group, biological replicates). Representative blots (**A**, **B**) and densitometric quantification are shown (**C**, **D**). [siTRIF = TRIF siRNA, siMyD88 = MyD88 siRNA, siCtrl = Ctrl siRNA].

Data information: Data are presented as mean ± SEM. One-way ANOVA followed by Dunnett's multiple comparisons test. ns = not significant; * *p* < 0.05.

**
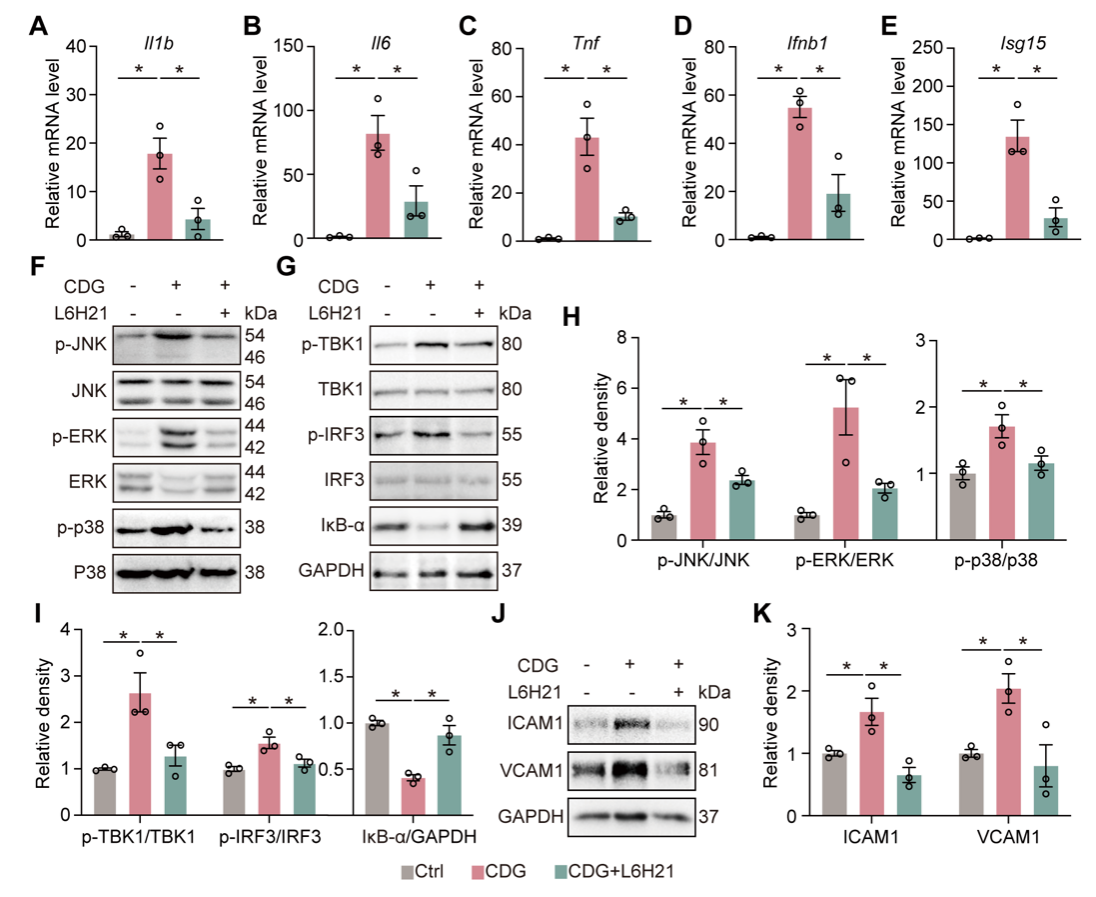
**

**Supplementary Figure S2. MD2 inhibitor prevents CDG-induced inflammatory responses in macrophages. A**-**E** MPMs were derived from WT mice. MPMs were pretreated with L6H21 (10 μM) for 2 hours and then exposed to CDG at a concentration of 80 μM for 12 hours. The mRNA levels of *Il1b*, *Il6*, *Tnf*, *Ifnb1*, and *Isg15* were measured (data normalized to *Actb*).

**F**-**I** MPMs were pretreated with L6H21 (10 μM) for 2 hours and then exposed to CDG (80 μM) for 1 hour. The protein levels of IκB-α, MAPK (ERK, JNK, and p38), TBK1, and IRF3 were determined using Western blot analysis. Unphosphorylated proteins and/or GAPDH were used as the loading controls. Representative blots (**F**-**G**) and densitometric quantification are shown (**H**-**I**).

**J**-**K** MPMs were pretreated with L6H21 (10 μM) for 2 hours and then exposed to CDG (80 μM) for 24 hours. The protein levels of ICAM1 and VCAM1 were examined by Western blot analysis. GAPDH was used as the loading control. Representative blots (**J**) and densitometric quantification are shown (**K**).

The data are presented as the mean ± SEM; n = 3 per group. A *p* value of < 0.05 was considered statistically significant; and * *p* < 0.05 assessed via ANOVA with the Bonferroni post hoc test for comparisons among more than two groups.


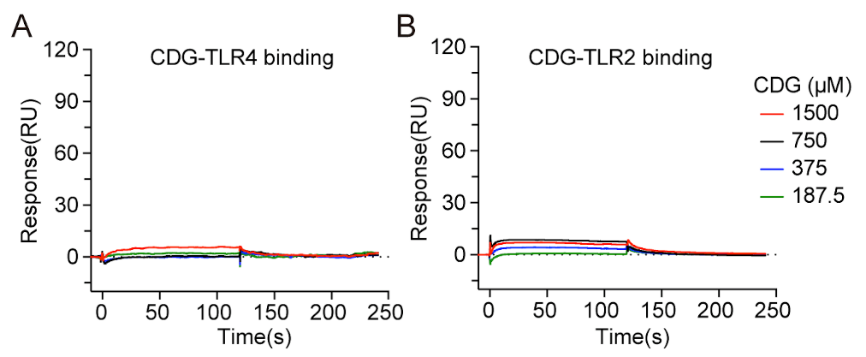


**Supplementary Figure S3.** The binding affinity of CDG to TLR4 (**A**) and TLR2 (**B**) human recombinant proteins was determined via surface plasmon resonance (SPR) analysis, respectively.

**
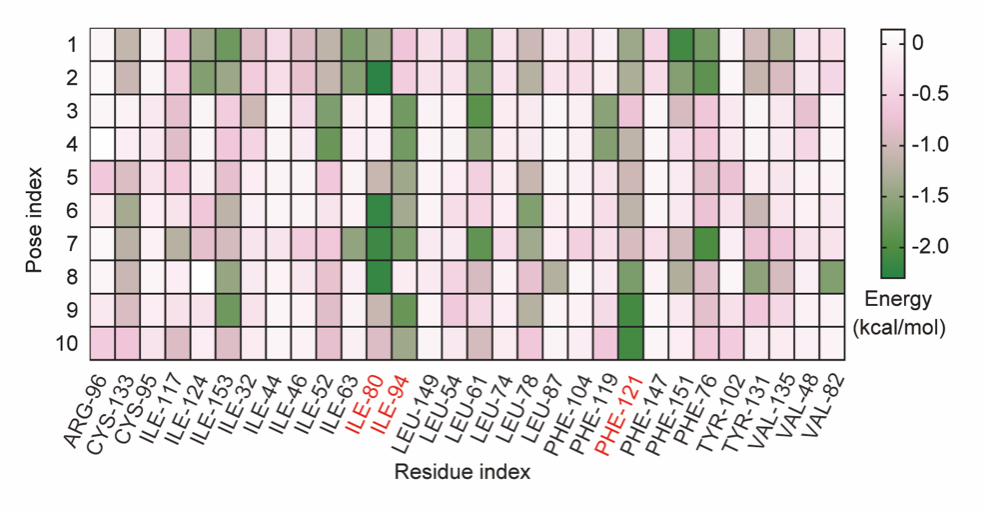
**

**Supplementary Figure S4.** Heatmap of binding energies for top 30 docking conformations of CDG-MD2 complex, with 10 poses each.

­
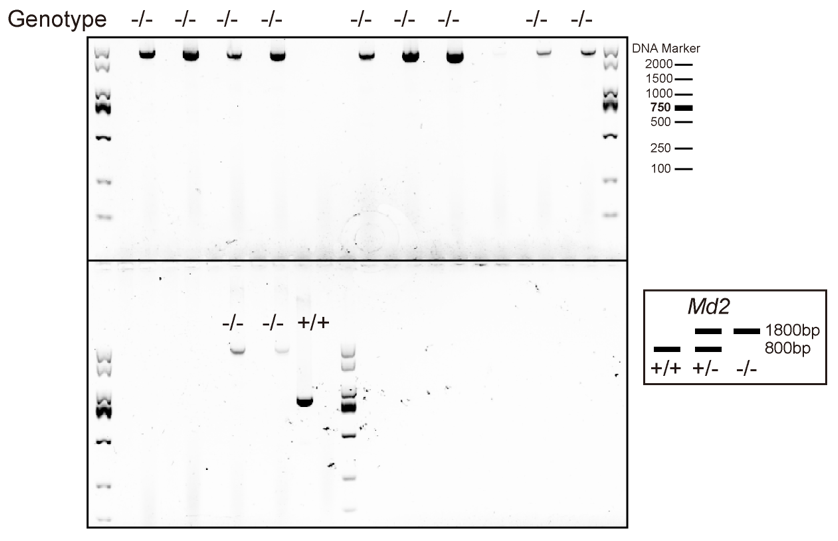


**Supplementary Figure S5.** The primers of *Md2* (WT: 800bp, KO: 1800bp) were used for PCR to identify the genotype of mice respectively. (-/-: *Md2* knockout mice; +/-: heterozygote mice; +/+: wildtype mice).


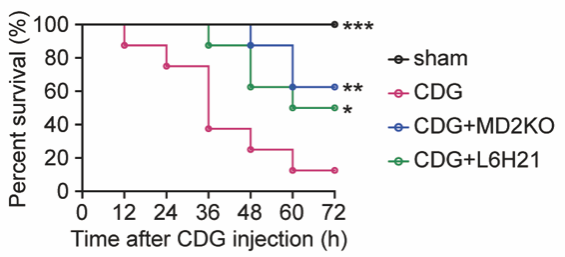


**Supplementary Figure S6.** **Survival curves of mice in the sham, CDG, CDG + MD2KO and CDG + L6H21 groups.** Sham: WT mice treated with vehicle control (0.9% saline). CDG: CDG (3 mg/kg, dissolved in 0.9% saline)-challenged WT mice. CDG + MD2KO: CDG-challenged MD2KO mice. CDG + L6H21: CDG-challenged mice treated with L6H21at the dose of 10 mg/kg.

Data information: Data information: survival data was analyzed by Log-rank (Mantel-Cox) test, *n* = 8 per group, *, *p* < 0.05; **, *p* < 0.01; ***, *p* < 0.001 *vs.* CDG group.

**
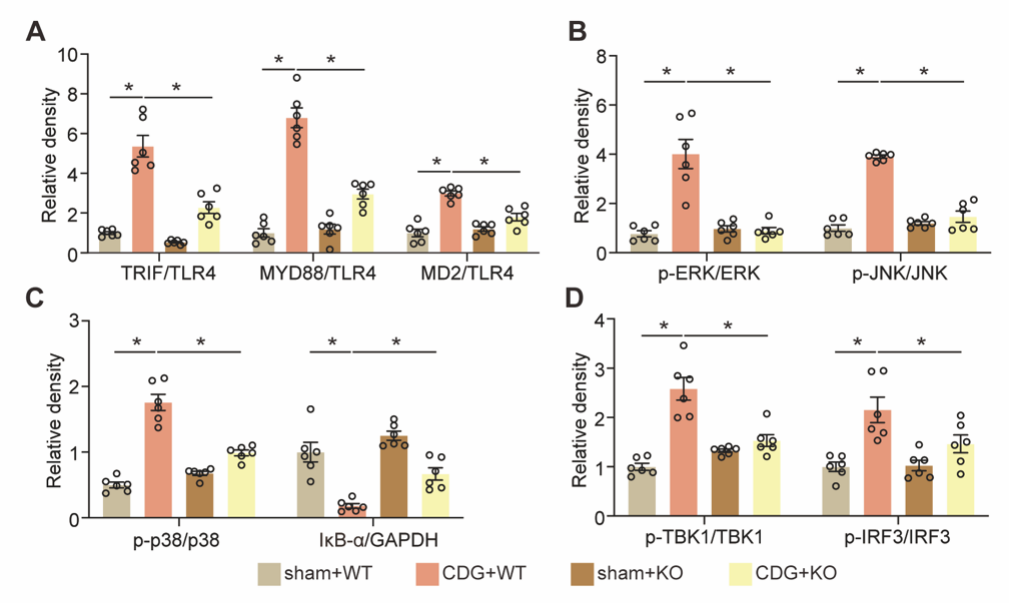
**

**Supplementary Figure S7. MD2 deficiency protects against CDG-induced inflammation *in vivo*.**

**A** C57BL/6J (WT) and MD2KO mice were challenged with intratracheal CDG (3 mg/kg) for 6 hours, to generate a lung injury model. Densitometric quantification for Fig. 6K.

**B**-**C** Densitometric quantifications for Fig. 6L.

**D** Densitometric quantifications for Fig. 6M.

Data information: data are presented as mean ± SEM, *n* = 6 per group. A *p* value of < 0.05 was considered statistically significant; and **p* < 0.05 assessed via ANOVA with the Bonferroni post hoc test for comparisons among more than two groups.
